# OmpR-Mediated Transcriptional Regulation and Function of Two Heme Receptor Proteins of *Yersinia enterocolitica* Bio-Serotype 2/O:9

**DOI:** 10.3389/fcimb.2018.00333

**Published:** 2018-09-20

**Authors:** Karolina Jaworska, Marta Nieckarz, Marta Ludwiczak, Adrianna Raczkowska, Katarzyna Brzostek

**Affiliations:** Department of Applied Microbiology, Institute of Microbiology, Faculty of Biology, University of Warsaw, Warsaw, Poland

**Keywords:** OmpR, Fur, *Yersinia enterocolitica*, HemR1, HemR2

## Abstract

We show that *Yersinia enterocolitica* strain Ye9 (bio-serotype 2/O:9) utilizes heme-containing molecules as an iron source. The Ye9 genome contains two multigenic clusters, *hemPRSTUV*-1 and *hemPRST*-2, encoding putative heme receptors HemR1 and HemR2, that share 62% amino acid identity. Expression of these proteins in an *Escherichia coli* mutant defective in heme biosynthesis allowed this strain to use hemin and hemoglobin as a source of porphyrin. The *hemPRSTUV*-1 and *hemPRST*-2 clusters are organized as operons, expressed from the p_hem−1_ and weaker p_hem−2_ promoters, respectively. Expression of both operons is negatively regulated by iron and the iron-responsive transcriptional repressor Fur. In addition, OmpR, the response regulator of two component system (TCSs) EnvZ/OmpR, represses transcription of both operons through interaction with binding sequences overlapping the −35 region of their promoters. Western blot analysis of the level of HemR1 in *ompR, fur*, and *ompRfur* mutants, showed an additive effect of these mutations, indicating that OmpR may regulate HemR expression independently of Fur. However, the effect of OmpR on the activity of the p_hem−1_ promoter and on HemR1 production was observed in both iron-depleted and iron-replete conditions, i.e., when Fur represses the iron-regulated promoter. In addition, a hairpin RNA thermometer, composed of four uracil residues (FourU) that pair with the ribosome-binding site in the 5′-untranslated region (5′-UTR) of *hemR1* was predicted by *in silico* analysis. However, thermoregulated expression of HemR1 could not be demonstrated. Taken together, these data suggest that Fur and OmpR control iron/heme acquisition via a complex mechanism based on negative regulation of *hemR1* and *hemR2* at the transcriptional level. This interplay could fine-tune the level of heme receptor proteins to allow *Y. enterocolitica* to fulfill its iron/heme requirements without over-accumulation, which might be important for pathogenic growth within human hosts.

## Introduction

*Yersinia enterocolitica*, a member of the genus *Yersinia* in the family *Enterobacteriaceae* is a human enteropathogen which causes yersiniosis, i.e., gut-associated diseases such as enteritis, diarrhea, and mesenterial lymphadenitis (Bottone, 1997). Multiple virulence factors encoded by chromosomal and plasmid pYV-located genes are involved in *Y. enterocolitica* virulence (Cornelis, 2002). In addition, iron-acquisition systems are considered important pathogenicity determinants. The concentration of iron in the environment is critical for the control of bacterial metabolism. Iron limitation in the host can abolish bacterial growth, whereas a high intracellular iron concentration may damage bacterial cells due to the formation of harmful reactive oxygen species (ROS). Thus, the transport, storage, and metabolism of iron have to be tightly controlled to maintain iron homeostasis (Hantke, 2001).

A variety of mechanisms are employed by *Y. enterocolitica* to take up iron from the host body, including siderophore-mediated uptake and systems for acquiring iron from abundant heme or hemoproteins (Caza and Kronstad, 2013). Pathogenic strains can be divided into two groups: those producing the siderophore yersiniabactin (biotype 1B) and those unable to produce this siderophore but able to use ectogenic siderophores released by other bacteria, such as ferrioxamin B and E or ferrichrome (biotypes 2–5) (Heesemann, 1987; Bäumler et al., 1993). About 70% of the iron in the human host is present within heme and/or hemoproteins (hemoglobin, myoglobin, cytochromes). A heme uptake system was identified previously in *Y. enterocolitica* bio-serotype1B/O:8 strain WA-C (Stojiljkovic and Hantke, 1992, 1994). This system, involved in the acquisition and transport of the entire heme moiety into the cytoplasm, consists of the receptor HemR, an ATP-binding cassette (ABC) transporter HemTUV, and a putative heme-degrading protein HemS. The energy for heme uptake is transferred from the inner to the outer membrane via the TonB/ExbB/ExbD system (Krewulak and Vogel, 2011). The protein TonB spans the periplasm and can physically interact with a highly conserved region of the receptor HemR called the TonB box. A conformational change in the heme receptor caused by this interaction permits the transport of the ligand across the OM into the periplasm (Nau and Konisky, 1989; Braun et al., 1991). Gene clusters responsible for the uptake and transport of heme have been identified in several bacterial species, including *Yersinia pestis* (*hmuRSTUV*) (Thompson et al., 1999), *Yersinia pseudotuberculosis* (*hmuRSTUV*) (Schwiesow et al., 2018), *Shigella dysenteriae* (*shu* genes) (Wyckoff et al., 1998), and *Vibrio cholerae* (*hut* genes) (Occhino et al., 1998), although their organization varies. The heme gene cluster of *Y. enterocolitica* bio-serotype 1B/O:8 strain WA-C contains six open reading frames (ORFs) *hemPRSTUV*, organized in an operon whose expression is enhanced by iron deprivation (Stojiljkovic and Hantke, 1992, 1994). To avoid high concentrations of free intracellular iron, the ferric uptake regulator (Fur) with Fe^2+^ cofactor efficiently represses the transcription of appropriate genes. Fur is a global transcriptional regulator that tightly controls the transport, storage, and metabolism of iron in many Gram-negative bacteria (Hantke, 2001). In addition, Fur represses the expression of genes involved in the regulation of multiple cellular functions such as the oxidative stress response, energy metabolism, acid tolerance, and virulence (Hassett et al., 1996; Ochsner and Vasil, 1996; Bijlsma et al., 2002; van Vliet et al., 2003).

Bacterial cells are constantly challenged by various environmental stresses in their natural habitats. *Y. enterocolitica* faces several different challenges during infection and colonization of the human body. Efficient adaptation to changing environmental conditions is possible due to the activity of sensory regulators such as TCSs, e.g., the EnvZ/OmpR signaling pathway. This prototype TCS, first identified and characterized in non-pathogenic *Escherichia coli* K-12, consists of the transmembrane histidine kinase EnvZ and the response regulator OmpR. EnvZ senses changes in the environment, undergoes autophosphorylation and then a phosphate group is transferred to OmpR. Conformational changes in the phosphorylated OmpR allow it to bind to DNA as a dimer and modulate gene expression (Kenney, 2002). EnvZ/OmpR is involved in the transcriptional regulation, in a positive or negative manner, of several genes/operons of *E. coli* in response to changes in osmolarity, pH, temperature, and the concentration of nutrients in the environment (Slauch and Silhavy, 1989; Higashitani et al., 1993; Shin and Park, 1995; Vidal et al., 1998; Yamamoto et al., 2000; Jubelin et al., 2005). OmpR was recognized as a pleiotropic regulator that controls the expression of genes involved in many different cellular processes such as chemotaxis, motility, drug sensitivity, or acid resistance, and virulence of pathogenic bacteria (Bernardini et al., 1990; Chatfield et al., 1991; Shin and Park, 1995; Bang et al., 2002; Feng et al., 2003; Stincone et al., 2011).

The relationship between virulence and the activity of the OmpR protein has also been described for *Y. enterocolitica* O:8 (Dorman et al., 1989) and *Y. enterocolitica* 2/O:9 (our studies). We have shown that OmpR is involved in the control of various cellular processes and functions in *Y. enterocolitica*, including adhesion, invasion, motility, Yop production, biofilm formation, and multidrug and serum resistance (Brzostek et al., 2003, 2007, 2012; Raczkowska et al., 2010, 2011a,b, 2015; Brzóstkowska et al., 2012; Skorek et al., 2013). Taken together, these findings have revealed the important role of OmpR in remodeling the cell surface and in the adaptation of *Y. enterocolitica* to different environmental niches, including the host body.

Comparative proteomic LC-MS/MS analysis of outer membranes prepared from *Y. enterocolitica* bio-serotype 2/O:9 strain Ye9 and its isogenic *ompR* deletion mutant AR4 identified HemR, an ortholog of enterobacterial heme receptor proteins, including HemR of *Y. enterocolitica* strain 8081 of bio-serotype 1B/O:8 (99% amino acid identity), as subject to negative regulation by OmpR. Moreover, preliminary data suggested an indirect role for OmpR in regulating *hemR* expression (Nieckarz et al., 2016).

Here, we demonstrate that the *Y. enterocolitica* Ye9 genome contains two multigene clusters, *hemPRSTUV*-1 and *hemPRST*-2, encoding homologous heme receptor proteins HemR1 and HemR2. Furthermore, we show that both clusters are organized as operons that are negatively regulated by Fur. More importantly, OmpR directly represses the expression of *hemR1* and *hemR2* through interaction with binding sequences located in the promoter regions of their respective operons. In addition, a zipper-like RNA structure closely resembling a FourU RNA thermosensor was recognized within the *hemR1* 5′-untranslated region (5′-UTR), but *hemR1* expression was not subject to thermoregulation at the post-transcriptional level. Our results suggest that OmpR controls HemR1 expression in both the absence and presence of the Fur repressor. Finally, we evaluated the importance of both heme receptor proteins in iron/heme acquisition by *Y. enterocolitica* and *E. coli* cells.

## Materials and methods

### Bacterial strains and growth conditions

The bacterial strains used in this study are described in Table S1. *E. coli* strains for plasmid manipulation and propagation were grown at 37°C in LB medium (10 g/l tryptone, 5 g/l yeast extract, 5 g/l NaCl). *E. coli* strain SASX77 (Δ*hemA* mutant), was grown in the presence of 50 mM aminolevulinic acid (ALA; Sigma-Aldrich) unless otherwise indicated. *Y. enterocolitica* strains were cultured at 26 or 37°C in LB medium. To achieve iron-depleted conditions, cultures were grown in LBD medium, i.e., LB supplemented with an inhibitory concentration of 150 μM 2,2′-dipyridyl (DPD; Sigma-Aldrich). Antibiotics were used at the following concentrations: nalidixic acid (Nal), 30 μg/ml; chloramphenicol (Cm), 25 μg/ml; kanamycin (Km), 50 μg/ml; gentamicin (Gm), 40 μg/ml; tetracycline (Tet), 12.5 μg/ml; spectinomycin (Sp), 100 μg/ml.

### Assay for utilization of heme-containing compounds as an iron source

*Y. enterocolitica* strain Ye9 grown under iron-depleted conditions was tested for the ability to obtain iron from heme-containing compounds. Initially, the growth yield (OD_600_) of this strain in LB supplemented with the iron chelator (DPD) to final concentrations of 0, 100, 150, 200, and 300 μM, was assessed. Cultures were incubated at 26 or 37°C for 28 h. Subsequently, heme and hemoglobin at final concentrations of 10 and 2.5 μM, respectively, were added to LBD (150 μM DPD) and the growth yield determined as above.

### Growth of *E. coli* strain SASX77 (Δ*hemA* mutant) carrying plasmids expressing *Y. enterocolitica* HemR1 or HemR2 proteins

The *E. coli* Δ*hemA* strain SASX77, which is defective in ALA synthetase, was transformed with the plasmid pACYC184 or its derivatives pHEM1 and pHEM2, and maintained on LB agar supplemented with 50 μM ALA and Tet. Overnight cultures of these strains were adjusted to an OD_600_ of 0.1 and 1 ml was harvested by centrifugation (2,400 × g, 2 min, RT). The pelleted cells were resuspended in 100 μl of 0.9% (w/v) NaCl, then mixed with 3 mL of 0.75% (w/v) agarose, and spread on LB agar plates. After solidification of the agarose, 5-mm diameter circles of Whatman filter paper (thickness 0.88 mm) were placed on the plates and these were wetted with 10 μl of different test solutions: 10 mM hemin, 0.1 mM hemoglobin, 0.9% (w/v) NaCl, or 50 mM ALA. The plates were photographed after incubation at 37°C for 48 h.

### CAS assay

Chrome azurol S (CAS) agar plates, prepared as described previously (Schwyn and Neilands, 1987; Neilands, 1994), were used to monitor siderophore production. CAS is an iron-dye complex that changes color from blue to orange when iron (Fe^3+^) is removed by the action of siderophores. Strains were grown overnight in LBD medium at 26°C and 10 μl aliquots were spotted onto the plates. The plates were photographed after incubation at 26°C for 48 h.

### Molecular biology techniques

All DNA manipulations, polymerase chain reactions (PCRs), restriction digests, ligations, and DNA electrophoresis, were performed as previously described (Sambrook and Russell, 2001). Plasmid and genomic DNA were isolated using a Plasmid Miniprep DNA purification Kit and Bacteria & Yeast Genomic DNA Purification Kit (EurX), respectively. When the amplified fragments were used for cloning, PCR was performed using DreamTaq DNA polymerase or Phusion High-Fidelity DNA polymerase (Thermo Scientific). Oligonucleotide primers for PCR and sequencing were purchased from Sigma Aldrich and are listed in Table S2. DNA fragments amplified by PCR were purified using a PCR/DNA Clean Up kit (EurX). The plasmids used in this study are described in Table S1. DNA sequencing was performed by Genomed S.A. (Warsaw, Poland).

### Semi-quantitative reverse transcription (RT)-PCR gene expression analysis

Cultures of *Y. enterocolitica* Ye9 were grown until OD_600_ ~1 in LBD medium at 37°C and then total RNA was isolated from 10^9^ cells using a High Pure RNA Isolation Kit (Roche). Following treatment with RNase-free DNase I (TURBO DNA-free^TM^ Kit, Invitrogen), the RNA was reverse-transcribed using Maxima H Minus reverse transcriptase (Thermo Scientific) primed with random hexamers. The cDNA was used as the template in PCRs (RNA as a negative control) with primer pairs RThPR1F/RThPR1R, RThRS1F/RThRS1R, and RThPR1F/RThPV1R (Table S2), specific for *hemPR*-1, *hemRS*-1, and *hemPRSTUV*-1, respectively, or RThPR2F/RThPR2R, RThRS2F/RThRS2R, RThRS2F/RThPV2R, and RThPR2F/RThPV2R specific for the *hemPR*-2, *hemRS*-2*, hemRST*-2, and *hemPRST*-2 mRNAs, respectively. The amplified fragments were mixed with RunSAFE stain (Cleaver Scientific), resolved by electrophoresis on 2% (w/v) agarose gels and visualized with a GE Healthcare AI600 Imager.

### Construction of transcriptional fusion plasmids p_hem-1_::*lacZ* and p_hem-2_::*lacZ*

To obtain transcriptional fusions of the p_hem−1_ and p_hem−2_ promoters with the *lacZ* gene, fragments of the *hemPRSTUV*-1 and *hemPRST*-2 operons containing the predicted promoters were first amplified from *Y. enterocolitica* Ye9 genomic DNA by PCR using the primer pairs hemP1F/hemP1R and hemP2F/hemP2R, respectively (Table S2). The obtained amplicons were digested with the restriction endonucleases EcoRI/KpnI and cloned into the corresponding sites of reporter vector pCM132Gm [derivative of plasmid pCM132 (Marx and Lidstrom, 2001) containing a Gm resistance cassette] upstream of a promoterless *lacZ* gene. The resulting constructs were verified by PCR using the primer pair pCM132GmSpr1/pCM132Spr2 (flanking the EcoRI and KpnI sites) followed by sequencing of the amplicons. The two constructs, named pCM1 (p_hem−1_::*lacZ*) and pCM2 (p_hem−2_::*lacZ*), were introduced into *E. coli* BW25113. These constructs were also introduced into *E*. *coli* S17-1 λ*pir* and transferred by conjugation into *Y. enterocolitica* Ye9N and the *ompR* mutant AR4. Selection of transconjugants was carried out on LB plates containing Gm and Nal (Ye9N) or Gm and Km (AR4). The presence of the plasmids in the *Y*. *enterocolitica* strains was confirmed by plasmid isolation and PCR with the primer pair pCM132GmSpr1/pCM132Spr2.

### Construction of a HemR1′-′GFP translational fusion

To measure post-transcriptional regulation of *hemR1* expression, a translational fusion with GFP was constructed in plasmid pFX-P (Schmidtke et al., 2013) using the Golden Gate technique (Engler et al., 2008). A DNA fragment carrying the 5′-UTR of *hemR1* plus the first 16 codons of the gene was amplified by PCR using the primer pair hemR-fw/hemR-rev and Ye9 genomic DNA as the template. In a separate PCR a fragment containing the *lacZ* promoter was amplified using primer pair OR181-plac-fw/OR182-plac-rv and plasmid pFX-P as template. The PCR primers contained BsaI sites and additional sequences designed to generate ends compatible with BsaI-cleaved vector pFX-P (Table S2). In a 20 μl Golden Gate cloning reaction, 40 fmol of vector were mixed with 40 fmol of each PCR product, 5 units of Eco31I (BsaI isoschizomer, Thermo Scientific), and 4.5 units of T4 DNA ligase (Thermo Scientific) in ligase buffer. The reaction was incubated at 37°C for 1 h, 5 min at 50°C, followed by 5 min at 80°C, and then it was used to transform *E. coli* BW25113. Clones were selected on LB agar supplemented with Sp and their identity confirmed by PCR and plasmid DNA sequencing.

### Construction of *fur* deletion mutants

The Δ*fur*::Gm deletion mutants of *Y. enterocolitica* Ye9N and the *ompR* mutant AR4 were obtained by homologous recombination using suicide vector pDS132 (Philippe et al., 2004). A *fur* gene mutated by the insertion of a Gm^R^ cassette was constructed by overlap extension PCR using primers listed in Table S2. As the first step, three DNA fragments were PCR-amplified using *Y. enterocolitica* genomic DNA (for flanking regions) or plasmid pBBR1MCS-5 Gm^R^ (for the Gm^R^ cassette) as the templates. Fragment A, extending from 403 bp upstream of *fur* to 6 bp within the ORF was amplified using primer pair Fur1/Fur2; fragment B, an 803-bp Gm^R^ cassette was amplified using primer pair Fur2/Fur3; fragment C, comprising the last 20 bp of the *fur* ORF plus 557 bp downstream of this ORF was amplified using primer pair Fur5/Fur6. A mixture of these three amplicons was then used as the template with flanking primers (Fur1/Fur6) in a PCR to generate the mutagenic fragment (1,860 bp). This was purified, digested with XbaI, and then cloned into the corresponding site of suicide vector pDS132. The resulting plasmid pDSfur was propagated in *E. coli* S17-1 λ*pir*, with selection on Cm and Gm, and then sequenced to confirm the absence of errors. This plasmid was introduced into two *Y. enterocolitica* strains, Ye9N and AR4, by biparental mating. Transconjugants created by single crossovers to integrate the allelic exchange plasmid into the Ye9N or AR4 genomes were selected in LB supplemented with Cm, Gm plus Nal (Ye9N), or Km (for AR4). Plasmid integration after a single crossover was verified by PCR. To force the second recombination, the transconjugant strains were plated on LB agar containing Gm and 10% (w/v) sucrose, and incubated at room temperature for 48 h. Sucrose-resistant colonies were screened for the loss of Cm resistance (encoded by the vector). The correct allelic exchange was verified for these *fur* mutants by PCR using the primer pair Fur0/Fur7 (Table S2) and by sequencing.

### Gel electrophoresis, preparation of cell extracts, and western blotting

For immunological detection of the HemR1 and HemR1′-′GFP proteins, *Y. enterocolitica* strains were grown under the desired conditions and extracts prepared from equal numbers of cells. The protein concentrations in the extracts were determined using the RC-DC protein assay (Bio-Rad) and if necessary they were diluted in Laemmli buffer to achieve equal loading (Sambrook and Russell, 2001). Samples were separated by electrophoresis on 12% TGX Stain-Free FastCast Acrylamide gels. Each gel was then Stain-Free activated by a 5 min exposure to UV and imaged using a GE Healthcare AI600 Imager. In some experiments, the loading of equivalent amounts of protein was controlled by Coomassie blue staining of an identical gel run in parallel. Next, the proteins were transferred to nitrocellulose membrane (Amersham Protran Western blotting membrane, nitrocellulose, pore size 0.2 μM; GE Healthcare) using a wet electroblotting system (Bio-Rad). The blots were probed with polyclonal rabbit antibodies against HemR1 (1:10,000, generous gift of Jürgen Heesemann) or GFP (Sigma Aldrich, 1:10,000). Sheep anti-rabbit IgG, conjugated to horseradish peroxidase (HRP, Promega) was used as the secondary antibody (1:15,000). Positive immunoreaction was visualized using Clarity ECL Blotting Substrate (Bio-Rad) for HRP-based chemiluminescent detection (GE Healthcare AI600 Imager).

### β-galactosidase assays

β-Galactosidase assays were performed essentially as described by Thibodeau et al. (2004), using 96-well microtiter plates (Nest Sc. Biotech.) and a Sunrise plate reader (Tecan). Briefly, cultures grown to stationary phase were diluted to an OD_600_ of 0.3–0.5 and 80 μl of each cell suspension were then mixed with 20 μl of POPCulture Reagent (EMD Millipore Corp) and incubated for 15 min to cause cell lysis. In the wells of a microtiter plate, 20 μl of each cell lysate were mixed with 130 μl of Z-Buffer and 30 μl of ONPG (4 mg/ml), as described by Miller (1992). For kinetic assays, the absorbance at 415 nm (relative to a blank) was measured at time intervals of 10 s, with 2 s of shaking before each reading. The assays were performed at 26°C and monitored for up to 20 min. The β-galactosidase activity was expressed in Miller units calculated as described previously (Thibodeau et al., 2004). Each assay was performed at least in triplicate.

### Electrophoretic mobility shift assays (EMSAs)

For *in vitro* DNA-binding studies, recombinant OmpR-His_6_ was expressed and purified as described previously (Nieckarz et al., 2016). Briefly, the N-terminal His-tagged OmpR protein (OmpR-His_6_, 29.78 kDa) was expressed from plasmid pETOmpR in *E. coli* BL21(DE3) and purified using Ni-NTA resin (Qiagen). The concentration of the purified OmpR protein was determined using the RC DC protein assay (Bio-Rad). DNA fragments comprising the regulatory regions upstream *hem*-1 and *hem*-2 clusters of the *Y. enterocolitica* Ye9 were amplified by PCR using primers listed in Table S2 with Ye9 genomic DNA as the template. The amplicons were purified using a Gene Matrix PCR/DNA Clean-Up kit (EurX) and the concentration of DNA was determined with a NanoDrop 2000. EMSA reactions (10 μl), contained 0.05 pmol of each DNA fragment, OmpR binding buffer (50 mM Tris-HCl pH 8.0, 100 mM KCl, 1 mM EDTA, 1 mM DTT, 20 mM MgCl_2_, 12% glycerol, 100 μg/ml BSA, 0.1% Triton X-100), and increasing amounts of OmpR-His_6_. After incubation at room temperature for 15 min, 2 μl of 30% (v/v) glycerol were added and the reactions were loaded onto a 4.2% native polyacrylamide gel (19:1 acrylamide/bisacrylamide, 0.2X TBE, 2% glycerol) that had been pre-run in 0.2X TBE running buffer for 40 min at 80 V. After loading, the electrophoresis was continued for 3 h at 110 V. The gels were then soaked in SYBRgreen 1X solution (Invitrogen) and visualized using a GE Healthcare AI600 imager.

### Sequence alignments and *in silico* modeling

Bioinformatic analyses examined the complete genome sequences of *Y. enterocolitica* subsp. *palearctica* Y11 and *Y. enterocolitica* subsp. *enterocolitica* 8081 (GenBank; http://www.ncbi.nlm.nih.gov/genbank/). Homology searchers were performed with BLAST software (https://blast.ncbi.nlm.nih.gov/Blast.cgi). Promoter prediction was conducted using the web-based software BPROM in the Softberry package (http://linux1.softberry.com/berry.phtml?topic=bprom&group=programs&subgroup=gfindb; Solovyev and Salamov, 2011). Predicted secondary structures of the partial and complete *hemR1* 5′-UTR were obtained using Mfold software (http://mfold.rna.albany.edu; Zuker, 2003). Western blot images were analyzed with Amersham Imager 600 Analysis Software V1.0.0 (GE Healthcare).

### Statistical analyses

Statistical analyses were performed using Prism 7 software (v. 7.02, GraphPad). One-way ANOVA was used to determine statistically significant differences.

## Results

### *Y. enterocolitica* Ye9 utilizes heme-containing molecules as an iron source

**Figure 1:**
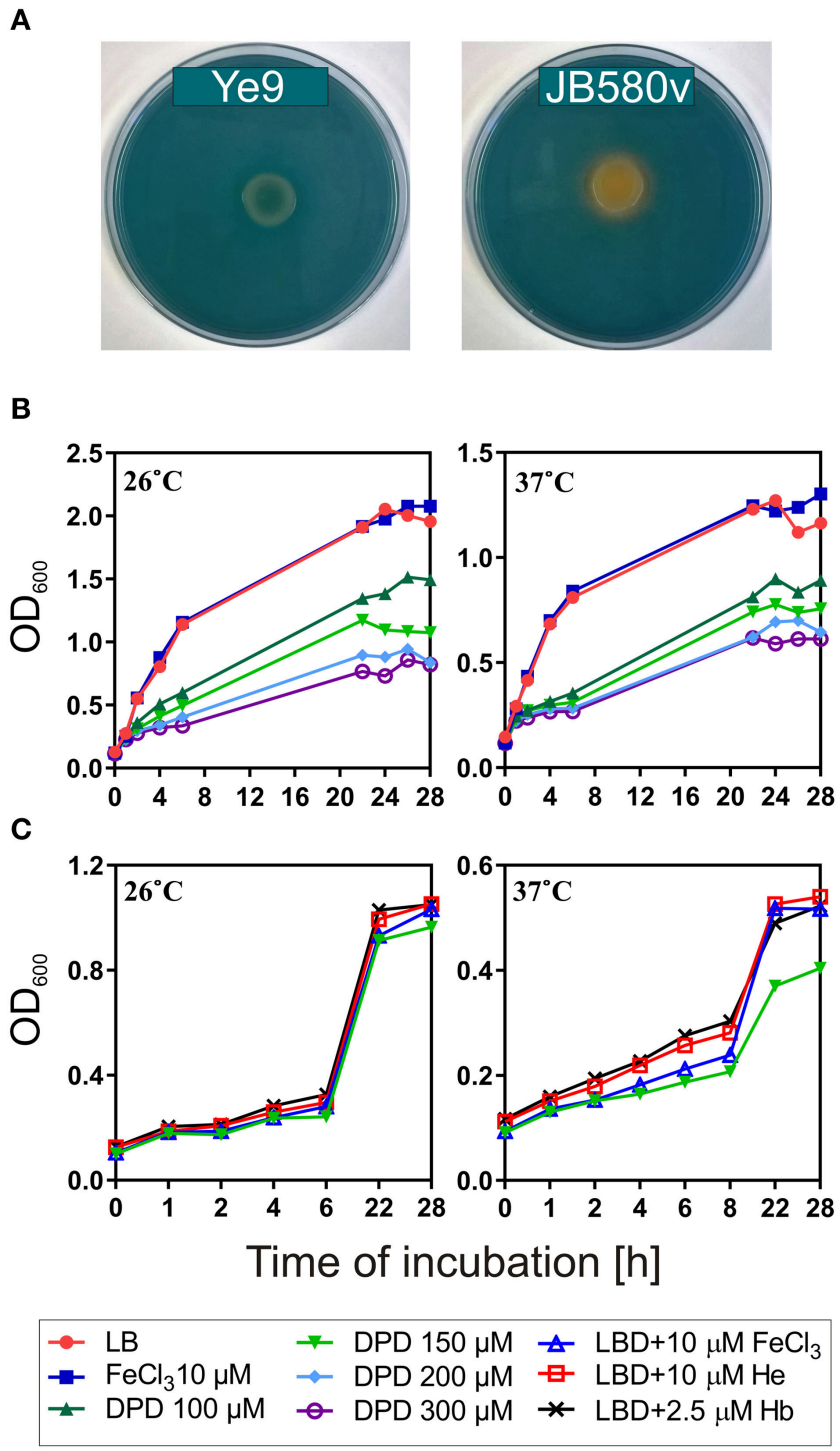
*Y. enterocolitica* Ye9 siderophore production and heme-containing molecule utilization. **(A)** Siderophore activity was assessed using CAS agar plates. No color change is seen on the plate carrying growth of Ye9. In contrast, a change in color from blue to orange is observed when iron is removed from the iron-CAS complex by the siderophore yersiniabactin produced by JB580v cells. **(B)** The growth yield of Ye9 determined by measuring the OD_600_ after 28-h incubation at 26 or 37°C in LB, LB with 10 μM FeCl_3_, LB with 100, 150, 200, or 300 μM 2,2′-dipyridyl (DPD). **(C)** The growth yield of Ye9 in LBD medium (150 μM DPD) containing hemin (He, 10 μM) or hemoglobin (Hb, 2.5 μM). The graphs present the results of one of two separate experiments performed in triplicate.

The wild-type strain Ye9 is a low-level pathogenic strain belonging to *Y. enterocolitica* bio-serotype 2/O:9. In contrast to more highly pathogenic *Y. enterocolitica* strains from bio-serotype 1B/O:8, it lacks the high-pathogenicity island (HPI), a chromosomal cluster of iron-regulated genes involved in the biosynthesis of siderophore yersiniabactin (Ybt) and the uptake of Fe-Ybt into cells (Pelludat et al., 1998; Carniel, 2001). To study the ability of *Y. enterocolitica* strains to produce siderophores, the strains Ye9 and JB580v (bio-serotype 1B/O:8) were spotted onto agar containing CAS and incubated at 26°C for 48 h (Figure 1A). As anticipated, an orange halo was observed around the colonies of JB580v, demonstrating its ability to synthesize yersiniabactin (Figure 1A), while Ye9 growth failed to produce any color change on the CAS plates, suggesting that it does not produce siderophores. Strain Ye9 was further characterized for its ability to utilize heme-containing molecules as an iron source. The growth yield of Ye9 incubated at 26 or 37°C in LB medium supplemented with 100–300 μM of the iron chelator 2,2′-dipyridyl (LBD) was significantly lower than in LB medium or LB with 10 μM FeCl_3_, and varied according to the chelator concentration (Figure 1B). However, the addition of hemin (He, 10 μM) or hemoglobin (Hb, 2.5 μM) caused moderate stimulation of the growth of Ye9 in LBD (150 μM 2,2′-dipyridyl) at 37°C, demonstrating that this strain can utilize heme-bound iron from hemin or hemoglobin (Figure 1C).

### Identification of two multigenic clusters encoding putative outer membrane heme receptor proteins HemR1 and HemR2 in the genome of *Y. enterocolitica* Ye9

**Figure 2:**
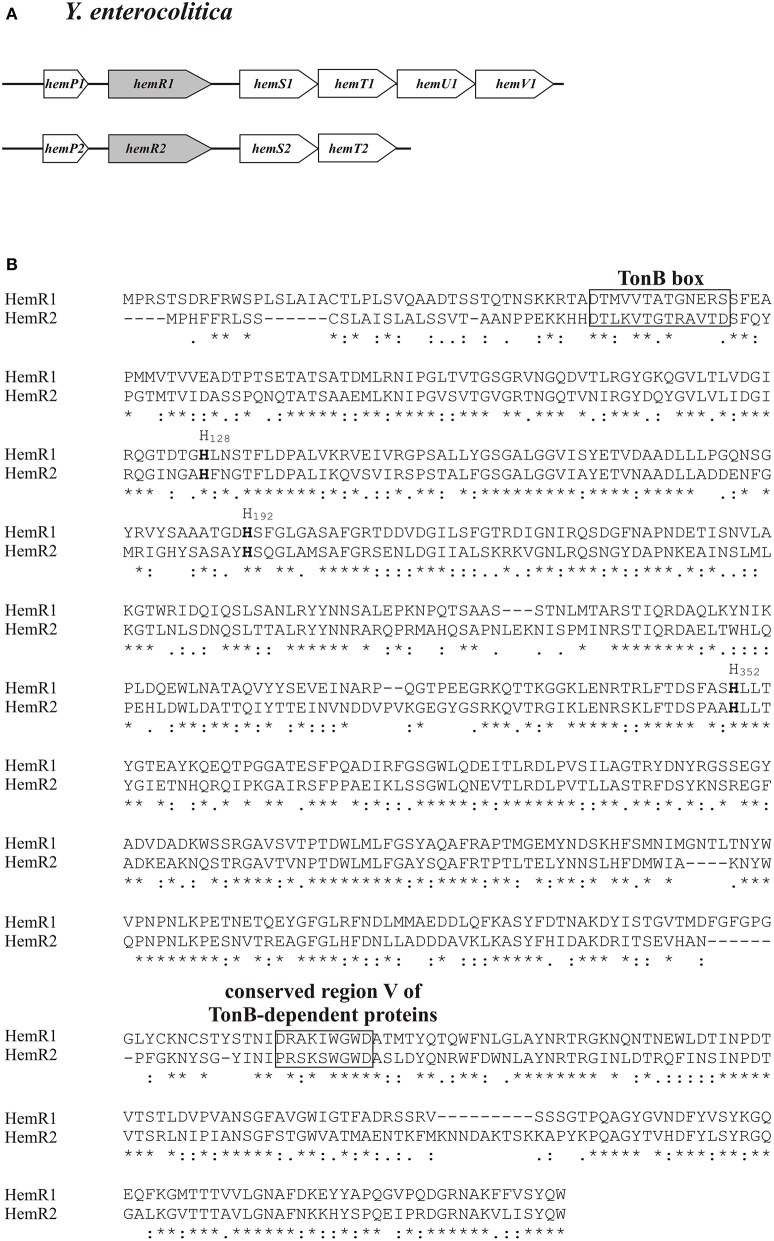
Organization of two heme uptake loci of *Y. enterocolitica* Ye9 and comparison of the encoded HemR homologs. **(A)** Genetic maps of heme transport loci 1 and 2. **(B)** Alignment of HemR1 and HemR2. The conserved histidine residues are in bold, and the TonB box and region V characteristic of all TonB-dependent OM proteins are boxed. Conserved amino acids are indicated by asterisks.

The *hemR* gene was previously recognized within the multigenic *hemPRSTUV* cluster responsible for the uptake and transport of hemin in highly pathogenic *Y. enterocolitica* (1B/O:8) strain WA-C (Stojiljkovic and Hantke, 1992). We established the presence of the *hemPRSTUV* cluster of *Y. enterocolitica* Ye9 (2/O:9) by PCR amplifications using primers designed from the Y11 (4/O:3) genome sequence (GenBank Acc. No. FR729477). Sequencing of these amplicons confirmed the presence of six ORFs *hemPRSTUV*-1 (*hem*-1 locus) (Figure S1). The sequence of this gene cluster was identical to that found in the Y11 (4/O:3) genome and it shared 99% sequence identity with *hemPRSTUV* of strain 8081 of bio-serotype 1B/O:8 (data not shown). The *Y. enterocolitica hemR1* gene encoding the outer membrane heme receptor protein HemR1 (687 amino acids) is situated downstream of the *hemP1* ORF encoding a small protein HemP1 (64 amino acids) of uncharacterized function (Figure 2A). The amino acid sequences of the HemR1 protein of *Y. enterocolitica* (2/O:9) strain Ye9 and *Y. enterocolitica* (1B/O:8) strain 8081 share 99% identity (data not shown). The deduced amino acid sequences of the proteins encoded by *hemSTUV*-1 in Ye9 are similar to the equivalent sequences of proteins of the 8081 strain. HemS1 is possibly involved in the release of iron from heme, and HemTUV-1 comprise subunits of a periplasmic/inner membrane ABC heme permease (Stojiljkovic and Perkins-Balding, 2002).

Bioinformatic analysis using BLAST software revealed the presence of a second multigenic cluster *hemPRST*-2 (*hem*-2 locus), encoding putative hemin transporter protein HemR2, in the genomes of *Y. enterocolitica* strains of low (2/O:9) and high pathogenicity (8081 strain, 1B/O:8) (Figure 2A). As for *hem*-1, we determined the sequence of the *hem-*2 locus in *Y. enterocolitica* strain Ye9 (Figure S1). Analysis of the structure of this novel heme transport locus revealed four intact ORF *hemPRST*-2 organized in an order similar to the hemin transport cluster *hem*-1. The predicted translation products of the *hem*-2 locus genes exhibit a high degree of amino acid sequence similarity to those encoded by the corresponding ORFs of *hem-*1 (HemP 52%, HemR 62%, HemS 53%, HemT 60% identity, respectively) (Figure S2, Figure 2B). The nomenclature for the *hem-*2 genes at locus 2 follows that of the highly homologous *hem* genes at locus 1, and the putative proteins encoded are named HemP2, HemR2, HemS2, and HemT2, respectively. Interestingly, the lack of *hemUV2* genes in this cluster suggests that the putative *hem*-2 heme permease is non-functional. This does not exclude the possibility that HemR2 might function as a receptor protein involved in heme uptake by utilizing components of the ABC transport system encoded by the *hem*-1 locus.

Alignment of HemR2 with the well-characterized HemR1 showed high amino acid sequence identity. These homologous proteins have typical TonB boxes at their amino-termini, conserved histidine residues, plus region V, which is characteristic of TonB-dependent proteins. These conserved motifs and residues were shown to be essential for heme transport through the receptor channel (Thompson et al., 1999) (Figure 2B).

### Genes within the *hemPRSTUV*-1 and the *hemPRST*-2 clusters are organized as operons

**Figure 3:**
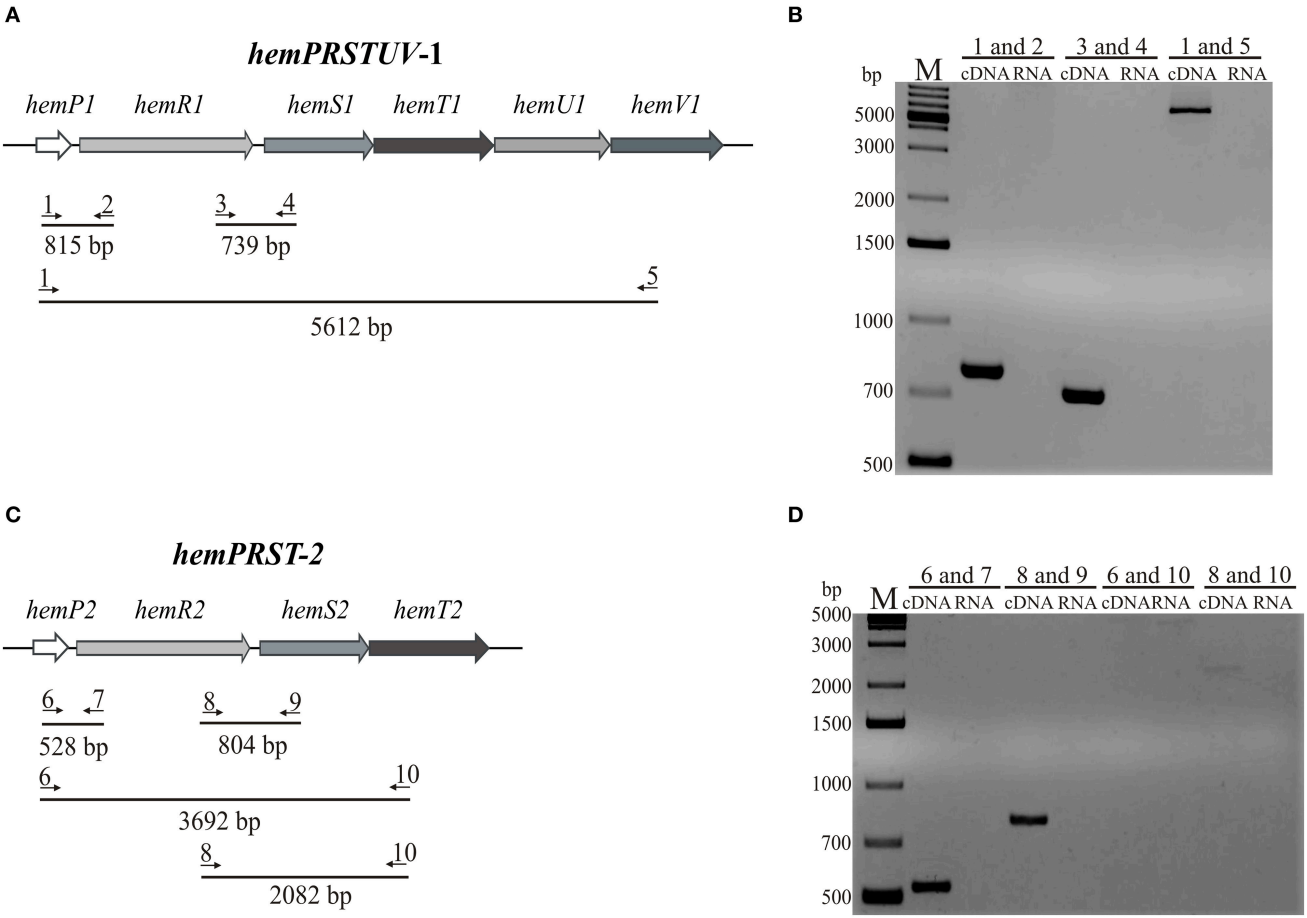
sqRT-PCR analysis of the *hemPRSTUV-*1 and *hemPRST*-2 gene clusters of *Y. enterocolitica* Ye9. **(A,C)** Scheme showing primers used in sqRT-PCR analysis of *hem* clusters 1 and 2. The sequences of the following primers are listed in Table S2. 1, RThPR1F; 2, RThPR1R; 3, RThRS1F; 4, RThRS1R; 5, RThPV1R; 6, RThPR2F; 7, RThPR2R; 8, RThRS2F; 9, RThRS2R; 10, RThPT2R. **(B,D)** Agarose gel electrophoresis of sqRT-PCR products from *hem* clusters 1 and 2. Total RNA isolated from strain Ye9 grown in iron-depleted LBD medium (LB with the iron chelator 2,2′-dipyridyl) at 37°C was DNase I treated and then reverse transcribed with random hexamers. The obtained cDNA was used as a template in PCR reaction with pairs of primers shown in **(A)** and **(C)**. RNA was used as the template in negative control reactions. M, GeneRuler 1 kb DNA Ladder.

There are six and four genes within the *hemPRSTUV*-1 (*hem*-1) and the *hemPRST-*2 (*hem*-2) clusters of *Y. enterocolitica* Ye9, respectively (Figure 2A). To determine whether the genes within these two loci might be transcribed as one transcriptional unit, sqRT-PCR analysis was performed on total RNA isolated from *Y. enterocolitica* Ye9 grown in iron-depleted medium (LBD). The locations of the primers used in this analysis are shown in Figures 3A,C and their sequences are given in Table S1. For the *hemPRSTUV*-1 cluster, the obtained RT-PCR products (815 and 739 bp) confirmed co-transcription of the *hemPR*-1 and *hemRS*-1 genes, respectively. The operon organization of *hemPRSTUV*-1 was conclusively demonstrated by the amplification of a 5,612-bp RT-PCR product (Figure 3B).

The operon organization of the *hemPRST-*2 cluster was also examined by sqRT-PCR analysis using the primers (Table S1) shown in Figure 3C. RT-PCR products of the expected sizes (528 and 804 bp) confirmed the co-transcription of the *hemPR*-2 and *hemRS*-2 genes, respectively. However, a 3,692-bp RT-PCR product spanning the entire *hemPRST*-2 operon could not be obtained, despite repeated attempts. Therefore, co-transcription of the *hemRST*-2 genes was tested. The presence of a faint RT-PCR product of the expected size (2,082 bp) confirmed the operon organization of the *hemPRST*-2 cluster (Figure 3D). Based on these data, it seems that the *hemPRST*-2 transcript might be particularly unstable or expressed at low levels at 37°C. Control PCRs with the tested primers and Ye9 genomic DNA as template gave positive results in all cases (data not shown).

### Role of HemR1 and HemR2 proteins in heme and hemoprotein utilization by *E. coli* Δ*hemA* strain SASX77

Previous studies have shown that a plasmid containing *hemPR*, part of the hemin operon of *Y. enterocolitica* WA-C O:8, permits an *E. coli* strain lacking a functional *hemA* gene to use hemin as a porphyrin source (Stojiljkovic and Hantke, 1992, 1994). To examine the importance of the two *Y. enterocolitica* Ye9 HemR proteins in the utilization of hemin and hemoglobin as sources of porphyrin, they were introduced into the *E. coli* Δ*hemA* strain SASX77, which is defective in ALA synthetase (auxotrophic for ALA, the heme biosynthetic precursor). The *hemPR* genes of the *hem*-1 and *hem*-2 operons with their native promoters were cloned into vector pACYC184 to obtain plasmids pHEM1 and pHEM2, respectively. These plasmids and the parent vector were introduced into *E. coli* SASX77. All transformant strains grew well in the presence of ALA, but no growth was observed on LB agar lacking this precursor.

**Figure 4:**
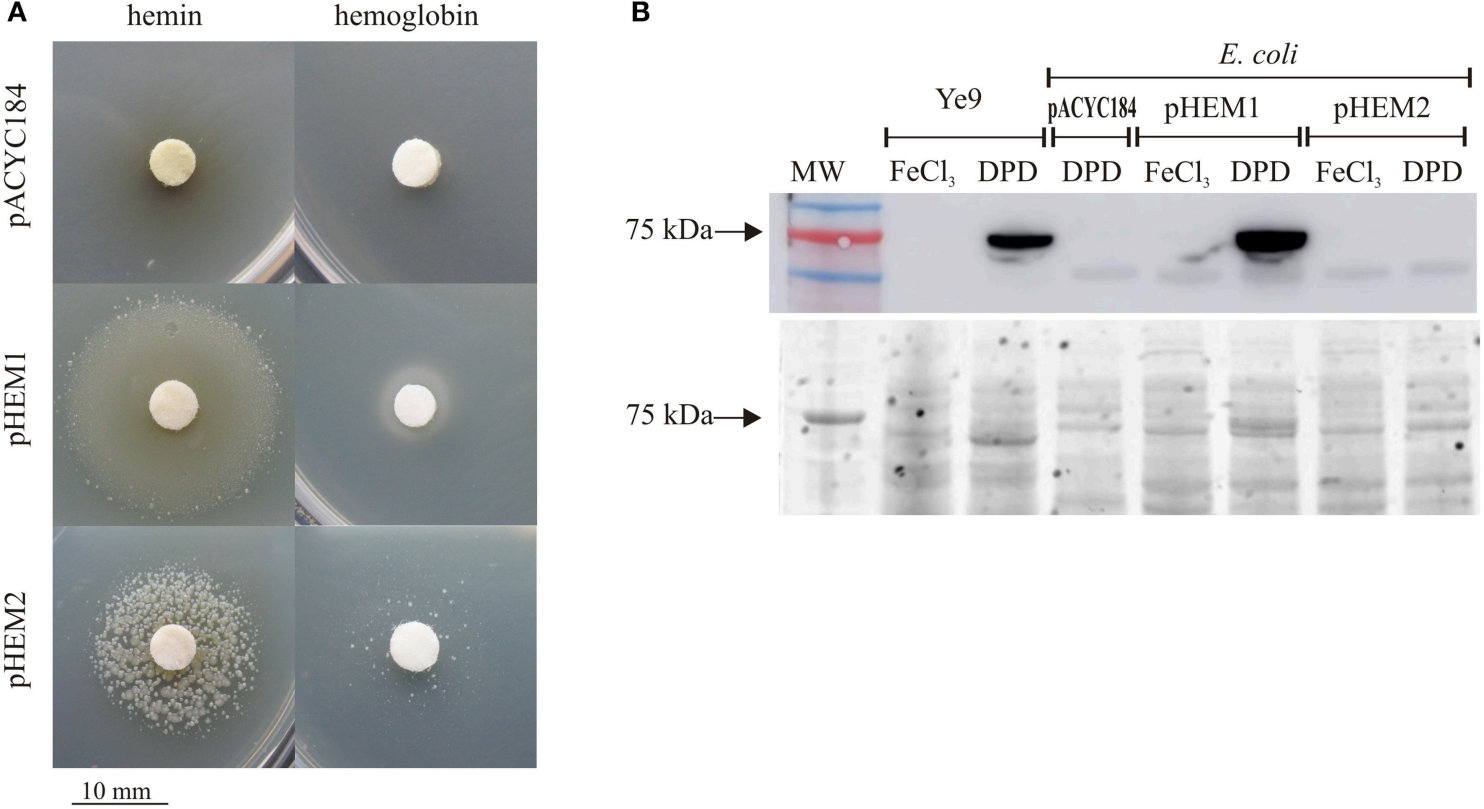
Growth promotion of *E. coli* Δ*hemA* strain SASX77 expressing receptors HemR1 or HemR2 in the presence of hemin and hemoglobin as porphyrin sources. **(A)** Ability of the *E. coli* Δ*hemA* strain expressing HemR1 or HemR2 to utilize hemin and hemoglobin. Diluted overnight cultures of SASX77/pHEM1 and SASX77/pHEM2 were mixed with agarose and overlaid on LB or LBD agar plates. Sterile paper discs wetted with 10 μl of hemin (10 mM) or hemoglobin (0.1 mM) were placed on these plates and zones of growth around these discs were analyzed after 1–4 days of incubation at 37°C. **(B)** Immunodetection of HemR1 in *Y. enterocolitica* Ye9N (wt) and *E. coli* SASX77 carrying plasmids pHEM1 (HemR^+^), pHEM2 (HemR2^+^), or pACYC184. Cell extracts from overnight cultures were analyzed by Western blotting with a polyclonal antibody directed against HemR1 **(upper)** and by TGX Stain free (**lower**, loading control). MW, 3-Color Prestained Protein Marker; kDa. This result is representative of at least three independent experiments.

To examine the effect of hemin or hemoglobin on the growth of strain SASX77 harboring plasmids containing *hemPR1* or *hemPR2*, a plate assay was used to detect utilization of these porphyrin sources in iron-replete (LB) and iron-deficient conditions (LBD). The strains expressing the receptors HemR1 or HemR2 were able to utilize exogenously supplied hemin and hemoglobin as porphyrin sources (Table 1, Figure 4). However, growth of the SASX77/pHEM1 (HemR1^+^) strain around the hemin and hemoglobin discs was significantly stronger than that of strain SASX77/pHEM2 (HemR2^+^). Moreover, the HemR2-expressing bacteria grew only as single colonies. Control strain SASX77/pACYC184 was unable to use either hemin or hemoglobin (Figure 4A). In iron-depleted conditions, the zone of growth stimulation was broad (up to 30 mm in diameter), but the colonies were very small and needed more time to develop (up to a week, in contrast to 1–2 days for the strains growing on LB) (data not shown). These results strongly suggest that both HemRs participate in the acquisition of heme and hemoproteins in *Y. enterocolitica* Ye9, and that HemR1 is significantly more efficient than HemR2.

**Table 1:** Assessment of HemR1 and HemR2 activity in hemin and hemoglobin transport in *E. coli* Δ*hemA* strain SASX77.

**Strain**	**Growth on**		
	**LB/Hemin**	**LB/Hemoglobin**	**LB/ALA**
SASX77/pACYC184	−	−	+++
SASX77/pHEM1	++	+	+++
SASX77/pHEM2	++^a^	++^a^	+++

−*No growth*.

+*~10 mm diameter growth zone around the discs*.

++*20–25 mm diameter growth zone around the discs*.

+++*>30 mm diameter growth zone around the discs*.

a *rowth as single colonies*.

Next, the level of HemR1 receptor protein in the *E. coli* strain carrying pHEM1 was determined by Western blot analysis with anti-HemR1 polyclonal antibody (Figure 4B). Cells of *Y. enterocolitica* Ye9 and of the *E. coli* strain carrying pACYC184 were used as a positive and negative control, respectively. All strains were grown in both iron-replete (LB+FeCl_3_) and iron-deficient (LBD) conditions. Extracts of cells grown to stationary phase at 37°C were immunoblotted and a strong HemR1 signal was produced by the samples from the Ye9 strain and *E. coli* DH5α/pHEM1 grown in LBD medium. As expected, no immunoreactive band was observed in the case of *E. coli*/pHEM2. This result indicated the absence of cross-reactivity between anti-HemR1 antibodies and the protein HemR2. Since no HemR2 antibody was available it was not possible to confirm expression of this protein in the *E. coli* strain carrying plasmid pHEM2. This experiment also demonstrated the induction of HemR1 protein in *Y. enterocolitica* grown under iron starvation conditions, confirmed that this gene can be expressed in a heterologous system from a recombinant plasmid, and showed that this expression is repressed by iron.

### OmpR inhibits the activity of the *hemPRSTUV*-1 and *hemPRST*-2 promoters

The putative promoters p_hem−1_ and p_hem−2_, located 42 and 43 bp upstream of the *hemP* gene in the *hemPRSTUV*-1 and *hemPRST*-2 operons, respectively, of *Y*. *enterocolitica* Ye9, were identified using the program BPROM (Solovyev and Salamov, 2011). Sequence analysis of the *hemPRSTUV*-1 and *hemPRST*-2 regions also led to the identification of an internal, putative promoter in the intergenic region between *hemR* and *hemS*. These findings corroborate the previously described locations of *hemPRSTUV* operon promoters in *Y. enterocolitica* WA-C (Stojiljkovic and Hantke, 1992), *Y. pestis* (*hmu* locus, Thompson et al., 1999), and *Y. pseudotuberculosis* (*hmu* locus, Schwiesow et al., 2018). *In silico* analysis revealed the presence of potential OmpR binding sites within the p_hem−1_ and p_hem−2_ promoters (see below), but not within the putative internal promoters, so their activity was not investigated further.

**Figure 5:**
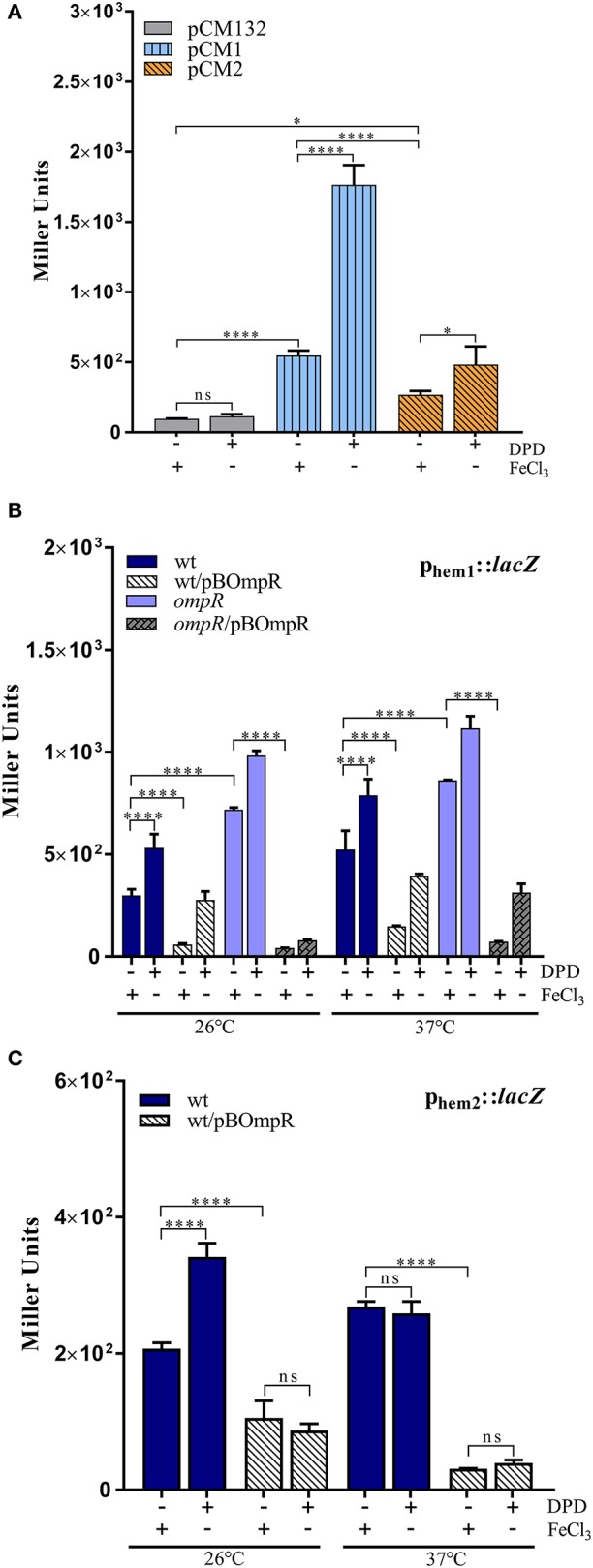
OmpR-dependent regulation of *hem*-1 and *hem*-2 operon expression. **(A**) Activity of the p_hem−1_ and p_hem−2_ promoters in *E. coli* BW25113 harboring the p_hem−1_::*lacZ* (pCM1) or p_hem−2_::*lacZ* (pCM2) fusion plasmids, compared with the same strain carrying parent vector pCM132. Strains were grown overnight in LB+FeCl_3_ or LBD medium at 37°C. **(B,C)** OmpR-dependent regulation of *hem*-1 and *hem*-2 expression in *Y. enterocolitica* strains grown to stationary phase in LB+FeCl_3_ or LBD medium at 26 or 37°C. **(B)** The wild-type Ye9N (wt), wt/pBOmpR, AR4 (*ompR*), and *ompR*/pBOmpR, harboring fusion p_hem−1_::*lacZ* (pCM1). **(C)** The wild-type Ye9N (wt) and wt/pBOmpR harboring fusion p_hem−2_::*lacZ* (pCM2). Plasmid pBOmpR carries the wild-type *ompR* allele. β-galactosidase activity was determined for each culture and expressed in Miller units. The data represent mean activity values with standard deviation from two independent experiments, each performed using at least triplicate cultures of each strain. Significance was calculated using one-way ANOVA [ns (non-significant) *P* > 0.05, ^*^P < 0.05, ^****^P < 0.00001].

To examine the activity of the p_hem−1_ and p_hem−2_ promoters and the influence of iron and the regulator OmpR, we generated transcriptional fusions of the putative promoter sequences with the *lacZ* gene in vector pCM132Gm. The resulting plasmids pCM1 (p_hem−1_::*lacZ*) and pCM2 (p_hem−2_::*lacZ*) were introduced into *E*. *coli* BW25113 and two strains of *Y. enterocolitica*: the wild-type strain Ye9N (both fusions) and the isogenic *ompR* mutant AR4 (only fusion p_hem−1_::*lacZ*). The transformed strains were grown to stationary phase in LB or LBD and β-galactosidase activity was measured (Figure 5).

In the *E. coli* strains grown at 37°C in both iron-replete or iron-depleted conditions the expression of the p_hem−1_::*lacZ* transcriptional fusion (pCM1) was significantly higher than that of p_hem−2_::*lacZ* (pCM2), indicating that p_hem−2_ is a weaker promoter. In addition, the expression of both p_hem−1_::*lacZ* and p_hem−2_::*lacZ* was upregulated in the presence of the iron chelator (~3-fold and ~2.5-fold, respectively), demonstrating inhibition by inorganic iron (Figure 5A).

To investigate whether the *hem*-1 and *hem*-2 operons are subject to OmpR-dependent regulation, p_hem−1_::*lacZ* and p_hem−2_::*lacZ* activity was measured in the *Y. enterocolitica* strains differing in the amount of OmpR grown at 26 or 37°C in iron-replete or iron-depleted conditions. With the p_hem−1_::*lacZ* fusion, a ~2-fold (26°C) and ~1.5-fold (37°C) up-regulation was observed under conditions of iron deficiency, confirming iron-dependent regulation of p_hem−1_. Moreover, β-galactosidase activity was increased in the absence of OmpR at both studied temperatures in iron-replete or iron-depleted conditions (Figure 5B). The effect of complementation of the *ompR* mutation in strain AR4 or overexpression of OmpR in the wild-type strain were studied by introducing plasmid pBOmpR carrying the wild-type copy of *ompR*. An increase in the level of OmpR caused a clear reduction in the expression of p_hem−1_::*lac*Z in cells grown in LB with FeCl_3_ or LBD, showing that OmpR negatively regulates expression of this fusion. These results indicated that *hem*-1 expression is controlled by an iron- and OmpR-dependent promoter located upstream of this operon. Both iron and OmpR participate in the negative regulation of *hem*-1.

To test the role of iron and OmpR in the regulation of p_hem−2_::*lacZ*, the wild-type strain Ye9N transformed with plasmid pCM2 was grown at 26 or 37°C in iron-replete or iron-depleted conditions. A ~1.5-fold increase in β-galactosidase activity was detected in wild-type cells grown at 26°C under iron-deficiency (Figure 5C). However, the inhibitory effect of iron on p_hem−2_::*lacZ* expression was not observed at 37°C, suggesting a more complex regulatory mechanism compared to the *hem*-1 promoter. Attempts to study the activity of the p_hem−2_::*lacZ* reporter fusion in the *ompR* mutant background were thwarted by the inability to transform strain AR4 with plasmid pCM2. Therefore, the wild-type *ompR* allele was introduced *in trans* (pBOmpR) into the Ye9N cells to monitor the effect of overexpression. The observed decrease in p_hem−2_ activity in the presence of *ompR* in multicopy in the wild-type strain grown at 26 or 37°C, suggested a negative role for OmpR in *hem*-2 expression. Interestingly, the inhibitory effect of iron was absent in the wild-type strain containing plasmid pBOmpR grown at 26°C (Figure 5C).

### Impact of fur and OmpR on HemR1 receptor production

**Figure 6:**
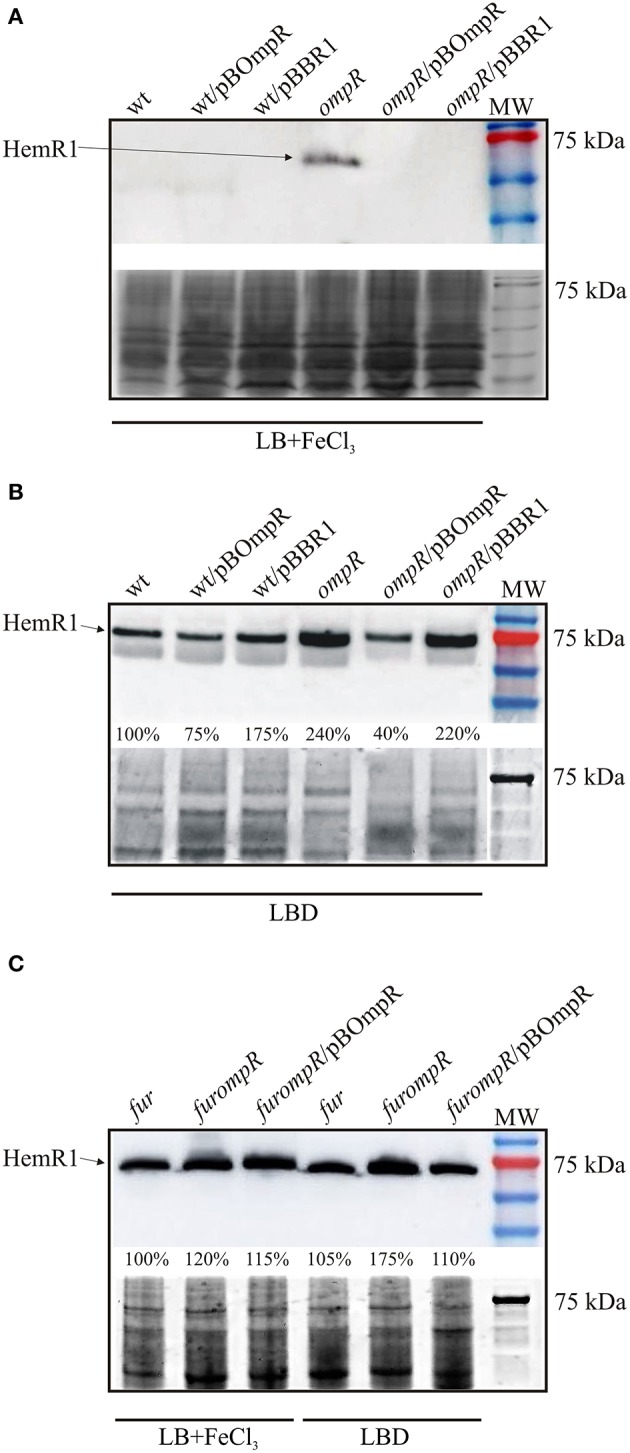
Effect of iron, Fur, and OmpR on HemR1 production. The level of HemR1 in cell extracts of strains grown overnight in LB+FeCl_3_ or LBD at 37°C analyzed by immunoblotting with a polyclonal HemR1 antibody. **(A,B)** The HemR1 level in the wild-type strain (wt), wt/pBBR1 (empty vector), wt/pBOmR (overexpression), the *ompR* mutant AR4, *ompR*/pBOmpR (complementation), and *ompR*/pBBR1 (empty vector) shown on Western blots **(upper)** with the stained gels as a loading control **(bottom)**. **(C)** The HemR1 level in the *fur* mutant background, i.e., *fur* mutant, *furompR* double mutant, and *furompR*/pBOmpR. The percentage values indicating the HemR1 band intensities in the tested strains relative to the wt **(B)** and *fur* mutant **(C)**, were determined using Amersham Imager 600 Analysis Software V1.0.0 (GE Healthcare). Stained gels shown as a loading control: **(A,C)** TGX Stain-Free gels (BioRad), **(B)** Coomassie blue stained gel. MW, 3-Color Prestained Protein Marker, kDa. The size of the HemR1 band is approximately 75 kDa on the Western blots. This result is representative of at least three independent experiments.

The analysis of strains carrying the *p*_*hem*−1_*::lacZ* reporter fusion construct suggested that iron and OmpR negatively influence the expression of the *hem*-1 operon at the transcriptional level. We next examined whether this regulatory effect was observable at the protein level. Western blot analysis with a polyclonal anti-HemR1 antibody was used to detect the HemR1 protein in cell extracts prepared from the following *Y. enterocolitica* strains: wild-type Ye9N, *ompR* mutant AR4, Ye9N/pBOmpR, AR4/pBOmpR, and strains with empty vector pBBR1. All strains were grown to stationary phase at 37°C in LB supplemented with FeCl_3_ (iron-replete) or LBD (iron-depleted). As shown in Figures 6A,B, HemR1 protein production was significantly influenced by iron content, because HemR1 was undetectable in strain Ye9N grown under iron-replete conditions, and highly abundant in iron-depleted conditions. Moreover, HemR1 production was notably increased in the *ompR* mutant compared to the wild-type strain in both media. Plasmid pBOmpR carrying the wild-type *ompR* allele, used to complement the *ompR* mutation in strain AR4, caused a clear reduction in the level of HemR1, confirming that OmpR negatively regulates its expression. In addition, the strain Ye9N/pBOmpR, which overexpresses OmpR, produced less HemR1 than strain Ye9N in LBD medium (Figure 6B). The presence of the empty vector pBBR1 in the wild-type strain and the *ompR* mutant, grown in LBD medium (Figure 6B) did not have any impact on the level of HemR1. However, for some unknown reason, a difference in the abundance of HemR1 was observed in the *ompR* mutant vs. the *ompR* strain carrying the empty vector pBBR1, when grown in iron-replete conditions (Figure 6A).

These data corroborated the results of the transcriptional fusion analysis and suggested that the Fur may be involved in iron-dependent inhibition of *hem*-1 operon expression, and thus HemR1 production.

In order to determine whether OmpR controls the level of HemR1 expression independently of the Fur repressor, the *fur* gene was deleted in both the wild-type strain Ye9N (Ye9*fur*) and the *ompR* mutant (AR4*fur*). HemR1 levels were then examined by immunoblotting of cell extracts prepared from the *fur, furompR* and *furompR*/pBOmpR strains grown to stationary phase in LB supplemented with FeCl_3_ or LBD at 37°C (Figure 6C). The Western blot data showed a high level of HemR1 production in the *fur* mutant background (independently of iron concentration), confirming that the Fur represses the biosynthesis of HemR1 in *Y. enterocolitica*. Interestingly, the strain also lacking OmpR (*furompR* mutant), grown in LBD medium, displayed a further increase in HemR1 protein abundance (ca. 70%), which suggested that OmpR inhibits the production of HemR1 independently of Fur. This effect was restored by complementation with plasmid pBOmpR carrying the wild-type *ompR* allele. Under iron-replete conditions the difference in HemR1 level between the *fur* and *furompR* mutants was less evident. These data suggested that iron may influence OmpR-dependent HemR1 synthesis irrespective of Fur.

### Interactions of OmpR with putative OmpR-binding sequences in the promoter regions of *hem*-1 and *hem*-2

**Figure 7:**
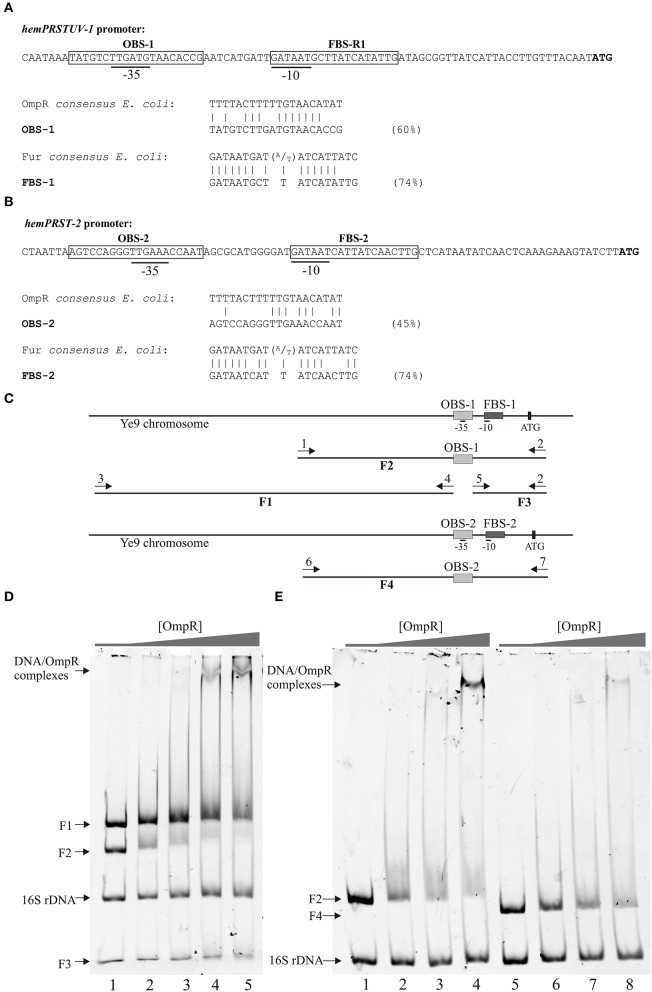
OmpR binding to fragments of the *hem*-1 and *hem*-2 promoter regions. **(A,B)** OmpR- and Fur-binding sites identified in promoter regions of the two heme uptake loci. The −35 and −10 promoter elements are underlined and putative regulator-binding sites are boxed. The ATG start codon of the *first* ORF of each operon is shown in bold. FBS, Fur-binding site; OBS, OmpR-binding site. **(C)** Schematic representation of fragments used in EMSA experiments presented in **(D)** and **(E)**. The numbered arrows represent the primers used to amplify the DNA fragments used in EMSAs: 1, hem1F; 2, hem1R, 3, hem1-aF; 4, hem1-aR; 5, hem1-bF; 6, hem2F; 7, hem2R. **(D)** EMSA with fragment F2 (326 bp) containing OBS-1 and neighboring fragments F1 (415 bp, upstream) and F3 (107 bp, downstream). A 16S rDNA fragment (211 bp) was used as a negative control. The reaction mixtures contained 0.05 pmol of each fragment and increasing amounts of purified OmpR-His_6_ were added: lane 1, no protein; lanes 2–5, 0.65, 1.31, 2.61, and 3.92 μM of OmpR-His_6_, respectively. **(E)** Binding of purified OmpR-His_6_ to fragments containing OBS-1 (F2, lanes 1–4) and OBS-2 (F4, lanes 5–8). The 16S rDNA fragment was used as a negative control. The reaction mixtures contained 0.05 pmol of each fragment and increasing amounts of purified OmpR-His_6_ were added: lanes 1 and 5, no protein; lanes 2 and 6, 0.65 μM; lanes 3 and 7, 1.31 μM; lanes 4 and 8, 3.92 μM.

We next tested whether OmpR influences *hem*-1 and *hem*-2 operon expression directly or if other regulatory factors are involved. *In silico* analysis of the *Y. enterocolitica* Ye9 *hem*-1 promoter region p_hem−1_, conducted using the consensus Fur binding site sequence of *E. coli* [FBS, 5′-GATAATGAT(A/T)ATCATTATC-3′] (Stojiljkovic et al., 1994; Escolar et al., 1999), led to the identification of the element FBS-1, with 74% homology to the *E. coli* FBS, that overlaps the −10 element of p_hem−1_ (Figure 7A). In addition, a putative OmpR binding site OBS-1, overlapping the −35 element of p_hem−1_ was identified by comparison with the consensus OmpR-binding sequence of *E. coli* (OBS, 5′-TTTTACTTTTTG(A/T)AACATAT-3′, Maeda et al., 1991). OBS-1 exhibits 60% identity to the *E. coli* OBS (Figure 7A).

*In silico* analysis of the *hem*-2 promoter region p_hem−2_ revealed a putative Fur binding site (FBS-2), as well as a potential OmpR binding site (OBS-2) in similar locations to FBS-1 and OBS-1. FBS-2 and OBS-2 are 74 and 45% identical to the *E. coli* consensus sequences, respectively (Figure 7B).

The ability of OmpR to directly interact with the predicted OBS elements was tested in electrophoretic mobility shift assays (EMSAs) using DNA fragments with or without the putative OmpR-binding sites (Figures 7C–E). Three p_hem−1_ fragments were PCR-amplified with the pairs of primers listed in Table S2. Fragment F1 (415 bp) represented a sequence upstream of OBS-1, F2 (326 bp) carried OBS-1, while F3 (107 bp) was a sequence downstream of OBS-1. When purified recombinant OmpR was mixed with equimolar amounts of these three p_hem−1_ fragments, shifted OmpR/DNA complexes started to appear at an OmpR concentration of 0.65 μM, and their amount increased as the concentration of the protein was raised (Figure 7D). The formation of these complexes was related to the disappearance of fragment F2, so were attributed to specific binding to OBS-1.

The interaction of OmpR with the putative OBS-2 within the promoter region of *hem*-2 was examined by incubating increasing amounts of recombinant OmpR with fragment F4 (303 bp), the region of p_hem−2_ containing OBS-2, or p_hem−1_ fragment F2 (326 bp), carrying OmpR binding site OBS-1. Specific OmpR/F4 complexes were observed at an OmpR concentration of 1.31 μM, with the simultaneous disappearance of fragment F4. In comparison, the appearance of OmpR/F2 complexes occurred at the lower OmpR concentration of 0.65 μM (Figure 7E). A 16S rDNA fragment (amplified with primer pair 16SF/16SR, Table S2), to which OmpR is unable to bind, was used as a negative control in both experiments. These results suggested that transcription of the *hem*-1 and *hem*-2 gene clusters is directly regulated by OmpR, and this regulation requires OmpR binding to specific sites in their promoter regions.

### The impact of temperature on post-transcriptional HemR1 expression

*Y. enterocolitica* HemR1 is a homolog (69% amino acid identity) of the *S. dysenteriae* ShuA receptor required for the utilization of heme as a source of iron (data not shown). Recently, thermoregulation of ShuA protein expression under iron-limited conditions was demonstrated, with low ShuA abundance at 25°C increased to high levels at 37°C (Kouse et al., 2013). Within the *shuA* 5′-UTR a regulatory element was recognized that closely resembles a FourU RNA thermometer: a zipper-like RNA structure that occludes the Shine-Dalgarno sequence at low temperatures, thus blocking translation of the *shuA* transcript.

Both HemR1 and ShuA are encoded within hemin transport loci, but the organization of these multigenic clusters differs considerably (Wyckoff et al., 1998). The *hemR1* gene of *Y. enterocolitica* is located 172 bp downstream of *hemP1*, the first gene of the *hemPRSTUV*-1 operon. There is no *hemP1* homolog in the hemin transport locus in *S. dysenteriae*. Moreover, the intergenic non-translated *hemP1-hemR1* region (172 nt) is present in polycistronic mRNA of *hem-*1. In comparison, the predicted promoter of *shuA* is located 328 bp upstream of the translation start site in a monocistronic transcript.

**Figure 8:**
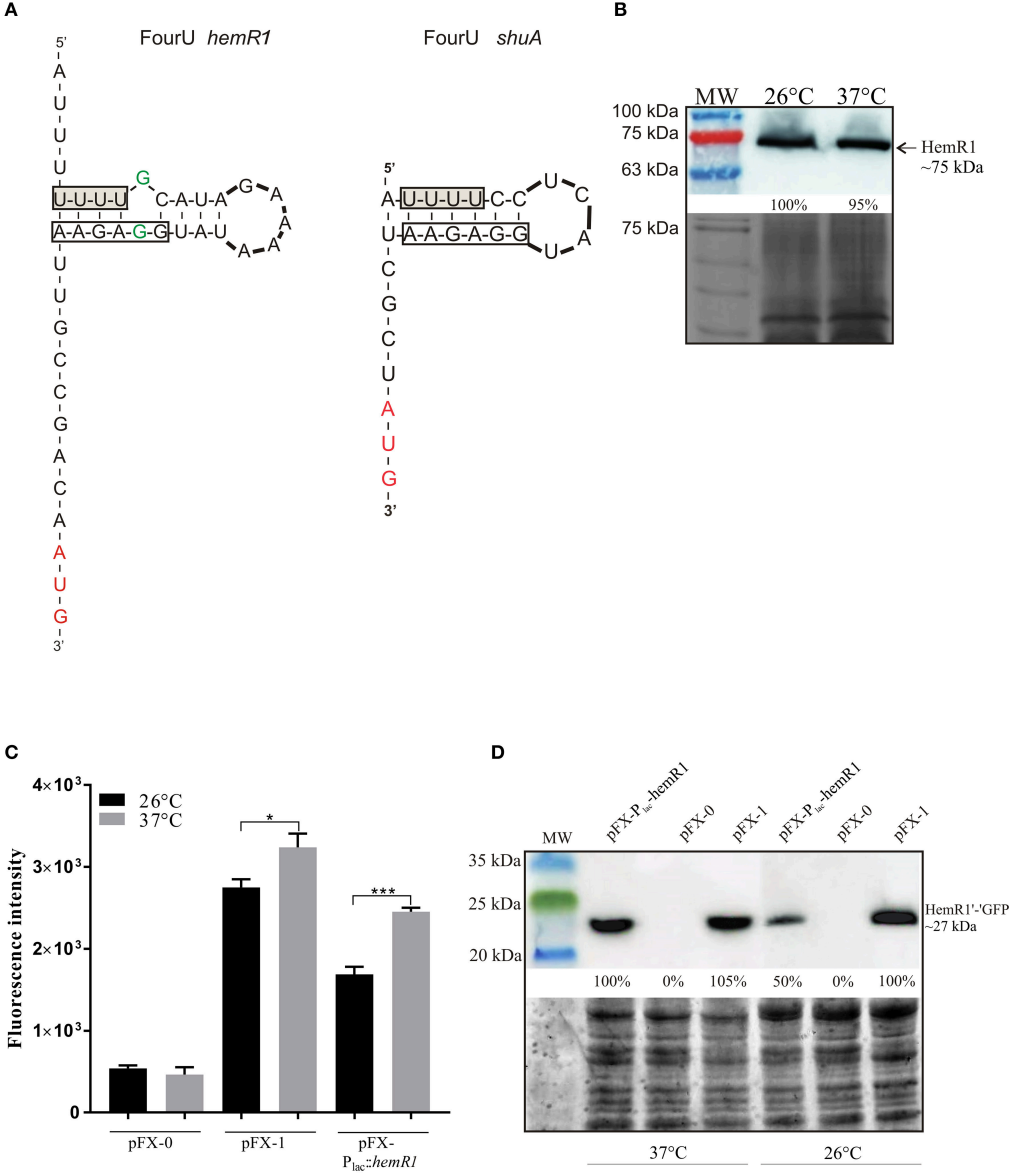
Influence of temperature on *hemR1* expression. **(A)** Potential FourU thermometer RNA secondary structures in the 5′-UTRs of *Y. enterocolitica* Ye9 *hemR1* and *S. dysenteriae shuA*, composed of a stretch of FourU and the ribosomal binding site (RBS), revealed by Mfold analysis (http://mfold.rna.albany.edu). Boxes indicate the location of the FourU motif (shaded) and putative RBS, the translation start codon is marked red, and unpaired nucleotides within the predicted secondary structure are in green. **(B)** Temperature-dependent HemR1 expression in *Y. enterocolitica* examined by immunoblotting. The analyzed samples were cell lysates of the *fur* mutant grown at 26 or 37°C in LB medium. The top panel shows the Western blot probed with a polyclonal antibody against HemR1, the bottom panel shows the Coomassie blue-stained gel as a loading control. The percentage values on the blot, indicating the HemR1 band intensities relative to that of cells grown at 26°C, were determined using Amersham Imager 600 Analysis Software V1.0.0 (GE Healthcare). **(C)** Expression of a *hemR1*′-′*gfp* translational fusion at different temperatures, monitored by fluorescence intensity. *E. coli* BW25113 harboring the reporter plasmid pFX-P_lac_-hemR1 or control plasmids pFX-0 and pFX-1 were grown in LB medium at 26 or 37°C. The GFP fluorescence intensity (RFU) of overnight cultures was determined. The data represent the averages ± SD from at least three experiments with duplicate cultures. Significance was calculated using one-way ANOVA [*P* > 0.05, ^*^P < 0.05, ^***^P < 0.001]. **(D)** GFP abundance examined by immunoblotting. The analyzed samples were cell lysates of *E. coli* BW25113 harboring the plasmids pFX-0, pFX-1 or pFX-P_lac_-hemR1. The top panel shows the immunoblot probed with antibody against GFP, the bottom panel shows the TGX Stain-Free gel as a loading control. MW, 3-Color Prestained Protein Marker; kDa. These results are representative of at least three independent experiments.

To investigate the possible thermoregulation of *hemR1* expression we analyzed an extended 5′-UTR of *Y. enterocolitica hemR1* for the presence of secondary structures using Mfold software (Zuker, 2003). This analysis identified a hairpin sequestering the *hemR1* ribosomal binding site, containing four consecutive uracil residues characteristic of a FourU RNA thermometer (Waldminghaus et al., 2007; Böhme et al., 2012). However, compared to the FourU base-pair region in the 5′-UTR of *shuA* there was moderate sequence variation in the surrounding regions (Figure 8A). No similar secondary structure was detected in the 5′-UTR of *hemR2* (data not shown).

The impact of temperature on HemR1 expression was assessed by immunoblotting of cell extracts prepared from the *fur* mutant following growth in LB medium to stationary phase at 26 or 37°C (Figure 8B). Identical levels of HemR1 were detected in the tested strain, irrespective of the growth temperature, which indicated that HemR1 production is not thermoregulated in *Y. enterocolitica* Ye9.

To further examine the potential thermoregulation of HemR1 expression at the post-transcriptional level, a plasmid pFX-P_lac_-hemR1, which carries the p_lac_ promoter linked to the 5′-UTR and first 16 codons of *hemR1* fused in frame with *gfp*, was constructed. Plasmid pFX-P_lac_-hemR1 and control plasmids pFX-0, containing promoterless *gfp*, and pFX-1, carrying *gfp* fused to the p_lac_ promoter (Schmidtke et al., 2013), were introduced into *E. coli* BW25113. Cultures of these strains were grown to stationary phase in LB medium at 26 or 37°C and green fluorescence intensity was measured (Figure 8C). Analysis of the strain carrying the *hemR1*′*-*′*gfp* fusion revealed a decrease in *hemR1* expression at 26°C relative to 37°C, however a slight reduction in fluorescence at this lower temperature was also observed for the control strain carrying pFX-1. In parallel, the impact of growth temperature on the GFP level was assessed by immunoblotting with an anti-GFP antibody (Figure 8D). The Western blot data corresponded well with the results of the fluorescence assay. A higher level of HemR1′-′GFP fusion protein was produced at 37°C compared to 26°C, which may indicate that temperature-dependent post-transcriptional regulation of *hem*-1 occur in *E. coli*. However, we could not exclude that observed effect may be explain also by the increased metabolic activity at the higher temperature.

## Discussion

The acquisition of iron/heme is necessary for pathogenic bacteria to grow within the host and to survive during the progression of an infection (Mietzner and Morse, 1994; Lee, 1995; Braun et al., 1998). On the other hand, both iron and heme are toxic at high concentrations (Anzaldi and Skaar, 2010). Thus, tight regulation of iron/heme acquisition is essential to fulfill the iron requirements of a pathogen while preventing harmful over-accumulation.

Studies on the role of OmpR in pathogenic *Yersinia* strains have provided evidence that this response regulator might be involved in the adaptation of yersiniae to diverse environmental conditions, which allows the bacteria to grow in distinct niches within and outside the host body (Brzostek et al., 2003, 2007; Raczkowska et al., 2011a,b, 2015; Brzóstkowska et al., 2012; Skorek et al., 2013). In a recent shotgun proteomic study we revealed that the regulator OmpR affects, both positively and negatively, the production of over 100 membrane proteins in *Y. enterocolitica* strain Ye9 of bio-serotype 2/O:9, a low pathogenicity strain that is not lethal for mice at low doses (Nieckarz et al., 2016). The loss of OmpR positively affected the production of heme receptor HemR1 in *Y. enterocolitica* cells grown in LB medium at 37°C and preliminary data suggested indirect repression of *hemR1* by OmpR.

In this study, we investigated the molecular mechanisms underlying OmpR-dependent production of two potential heme receptor proteins encoded within *hemPRSTUV*-1 (*hem*-1 locus) and *hemPRST*-2 (*hem*-2 locus) gene clusters identified in *Y. enterocolitica* strain Ye9. Firstly, we demonstrated that strain Ye9 utilizes heme-containing molecules as an iron source. The *Y. enterocolitica* hemin uptake system involved in transporting the entire heme moiety into the cytoplasm was described previously in strain WA-C, a mouse-virulent strain of bio-serotype 1B/O:8 (Stojiljkovic and Hantke, 1992, 1994). In contrast to strain Ye9, this strain is able to produce and utilize the siderophore yersiniabactin (Ybt), encoded on a chromosomal HPI (36 kb) (Heesemann et al., 1993; Rakin et al., 1994, 2012; Pelludat et al., 1998).

Heme uptake systems have been identified in several pathogenic bacteria, including *Y. pestis* (Thompson et al., 1999), *Y. pseudotuberculosis* (Schwiesow et al., 2018), *S. dysenteriae* (Mills and Payne, 1995, 1997; Wyckoff et al., 1998), and *V. cholerae* (Henderson and Payne, 1994; Occhino et al., 1998). Partial redundancy of heme-acquisition systems is often observed in Gram-negative bacteria due to the presence of uptake systems with a specific outer membrane receptor for heme and heme-proteins and/or the production of secreted hemophores (Ochsner et al., 2000; Runyen-Janecky, 2013).

In this study, two multigenic clusters, *hemPRSTUV*-1 and *hemPRST*-2 were identified in the genome of Ye9. The genetic organization of these clusters is identical except that the *hem*-2 locus lacks two ORFs, *hemU2* and *hemV2*, encoding potential components of the heme transport system. Evidence from sqRT-PCR analysis revealed that both the *hem*-1 and *hem*-2 clusters are organized as operons, and expressed from the promoters p_hem−1_ and p_hem−2_ located upstream of the respective *hemP* genes. Experiments with a *Y. enterocolitica* strain carrying a plasmid-located p_hem−1_::*lacZ* reporter fusion showed that p_hem−1_ promoter activity is inhibited by iron, which suggested the involvement of Fe^2+^-Fur in this repression. It has been previously demonstrated in diverse microorganisms that Fur, activated upon binding ferrous iron, binds to specific genes to inhibit their transcription (Bagg and Neilands, 1987; de Lorenzo et al., 1987; Andrews et al., 2003). Based on experimental and/or bioinformatic analyses, almost all iron-acquisition systems of pathogenic yersiniae, i.e., *Y. pestis*, have been shown to be Fur-controlled (Gao et al., 2008). More importantly, the results we obtained using transcriptional fusions with the *lacZ* gene and by Western blot analysis demonstrated that OmpR negatively regulates p_hem−1_ activity and thus HemR1 protein expression under conditions of iron starvation. The precise role of OmpR in the repression of HemR1 expression was revealed in a *fur* mutant background. While the *ompR* and *fur* mutations alone led to an increase in HemR1 synthesis, the combination of the two produced an additive effect. These data showed that OmpR inhibits HemR1 production in the absence of Fur. However, the OmpR-dependent regulation of HemR1 expression seems to be much more complex, since the effect of OmpR was also observed at the transcriptional and HemR1 protein level in iron-replete conditions, when the Fur repressor is active.

EMSA analysis revealed that OmpR acts directly at the p_hem−1_ promoter. We observed that OmpR specifically recognizes and binds a p_hem−1_ promoter fragment containing the *in silico-*predicted OmpR-binding sequence. This result confirmed a direct role for OmpR in mediating *hemR1* repression. In the *hem*-1 promoter, the predicted 19-bp Fur box (FSB-1) overlaps the −10 promoter motif, and is only 10 bp downstream of the 20-bp putative OmpR-binding site (OBS-1) at the −35 motif. Based on the location of these binding sites, we propose the following model for Fur- and OmpR-dependent repression of *hem*-1. Consistent with the notion that under iron-starved conditions Fur-dependent repression is abolished, OmpR may interact with OBS-1 and prevent the polymerase binding to the promoter, leading to inhibition of *hem*-1 expression. In iron-replete medium, there are a number of possible ways in which OmpR could regulate *hem*-1 expression. It is likely that binding of OmpR to the p_hem−1_ promoter leads to a DNA structure that is favorable for the function of Fe^2+^-Fur. Recent reports have suggested that the OmpR of *Salmonella enterica* may influence DNA topology to control target promoter activity (Cameron and Dorman, 2012; Quinn et al., 2014). We do not rule out the possibility that OmpR-Fur protein interactions could stimulate the binding of Fur to the *hem*-1 promoter, which would lead to inhibition of transcription initiation. Further research is required to characterize the molecular mechanisms modulating the activity of Fur at the *hem*-1 promoter in the presence of OmpR.

Similarly to the *hem*-1 locus, the *hem*-2 gene cluster *hemPRST*-2 is organized as an operon. However, p_hem−2_ promoter activity was much lower than p_hem−1_ and iron repression was detected only at 26°C. Although a sequence displaying 74% identity to the Fur box consensus was recognized in p_hem−2_ (at the −10 motif), the potential regulatory role of Fe^2+^-Fur might be dependent on other thermoregulated factors. The role of OmpR in the activity of p_hemR2_ was studied by analyzing the expression of a p_hem−2_::*lacZ* fusion in the wild-type strain with a normal or raised OmpR content. This experiment revealed that OmpR negatively regulates *hem*-2 expression. Interestingly, in the presence of an increased level of OmpR, iron regulation was abolished. Moreover, in EMSAs, we were able to detect the specific interaction of OmpR with the putative OmpR binding site in p_hem−2_, but with lower affinity than with OBS-1 in the *hem*-1 promoter. The higher binding sequence degeneracy in p_hem−2_ (45% identity to the consensus sequence for OBS-2 vs. 60% identity for OBS-1) might explain the weaker interaction of OmpR with this promoter.

Taken together, these results demonstrated the importance of the OmpR regulator in *hem*-1 and *hem*-2 expression, and hence the production of the HemR1 and HemR2 heme receptors in *Y. enterocolitica*. Our findings suggest that the effects of OmpR on *hem*-1 and *hem*-2 transcription are likely to be direct, i.e., produced by binding to specific DNA sequence elements in the promoter regions. The negative effect of OmpR on *hem*-1 transcription occurs in both the absence and presence of the Fur repressor. The role of OmpR in regulating *hem*-2 expression is difficult to assess and needs further study.

Thermo-induced structural changes in mRNA play a fundamental role in temperature sensing in bacteria and influence virulence gene expression in many pathogens, including those of the genus *Yersinia* (Narberhaus et al., 2006; Narberhaus, 2010; Böhme et al., 2012). Analysis of the intergenic region of the *hemP1-hemR1* transcript in locus *hem-*1, but not in locus *hem*-2, in *Y. enterocolitica* Ye9, suggested the formation of a secondary structure in the *hemR1* 5′-UTR with a FourU element that may sequester the ribosome binding site. Such an RNA structure was previously shown to be sufficiently stable at moderate temperatures (25°C) to inhibit expression of the ShuA receptor in *S. dysenteriae*. Melting of the FourU motif at 37°C permits access of ribosomes and initiates *shuA* translation (Kouse et al., 2013). However, Western blot analysis revealed that HemR1 of *Y. enterocolitica* is not subject to thermoregulation. Despite the presence of a FourU sequence in the 5′-UTR of *hemR1*, the unpaired G-G sequence within the hairpin may destabilize this secondary structure. In *S. dysenteriae*, only a single nucleotide replacement within the *shuA* hairpin led to destabilization of this inhibitory structure and resulted in increased expression of this gene at the non-permissive temperature of 25°C (Kouse et al., 2013). A stretch of four uracils located within an intergenic region of the *yscW-lcrF* transcript in a pathogenic *Yersinia* has been linked with the thermally regulated expression of the transcript encoding LcrF, the transcriptional activator of *yop* and *ysc* genes of the type III secretion system (Böhme et al., 2012). It is notable that analyses of the expression of a *hemR1*′*-*′*gfp* translational fusion and the level of a HemR1′-′GFP fusion protein in *E. coli* showed a degree of temperature dependence, suggesting that a mechanism of thermoregulation, unconnected with the presence of a FourU RNA thermometer, is active in this heterologous genetic background.

HemR1 and the newly identified HemR2 protein exhibit a high degree of amino acid sequence similarity (62% identity), including the presence of signature motifs like the TonB box, the conserved V region associated with all TonB-dependent OM proteins, and histidine residues required for heme transport via the receptor channel (Kadner, 1990; Bracken et al., 1999). Significant similarities between HemR1/HemR2 and hemin-binding proteins suggest that these two *Y. enterocolitica* proteins are involved in the binding and utilization of hemin and heme-proteins. The expression of the HemR1 or HemR2 protein from their native promoters in *E. coli* SASX77, a Δ*hemA* mutant defective in the biosynthesis of a heme precursor and naturally lacking a heme binding protein in the outer membrane (Stojiljkovic and Hantke, 1994; Mills and Payne, 1995), permitted hemin and hemoglobin utilization. The strain expressing HemR2 was less efficient in utilizing hemin or hemoglobin, which might reflect lower activity of the p_hem−2_ promoter. It is also possible that structural or functional differences in these OM receptors may influence the transport of the heme moiety.

Taken together the findings of this study indicate that *Y. enterocolitica* HemR1 and HemR2 are outer membrane-receptors that play an important role in hemin and hemoglobin utilization. We hypothesize that HemR1 and HemR2 together with HemTUV-1, a periplasmic/inner membrane ABC heme transporter also encoded by the *hem*-1 locus, may constitute a system involved in the acquisition of the heme moiety from host hemoproteins under the varied conditions encountered by *Y. enterocolitica* during an infection. It is also possible that HemR2 together with an as yet unidentified cytoplasmic transporter constitute an alternative system for the utilization of heme and host hemoproteins. Such a system might be able to counteract the loss/inactivation of the *Y. enterocolitica* heme uptake system based on the HemR receptor. Pathogenic bacteria frequently possess more than one transport system for iron or heme/hemoprotein uptake (Braun et al., 1998), so additional systems besides those involving the TonB-dependent HemR1 and HemR2 receptors may help perform this vital function in *Y. enterocolitica*.

In conclusion, this study has demonstrated that the regulators Fur and OmpR participate in a complex mechanism governing the negative regulation of *Y. enterocolitica hemR1* and *hemR2*. This interplay might be responsible for fine-tuning the expression of HemR receptor proteins mediating iron/heme acquisition during infection, to permit rapid growth while avoiding toxicity.

## Author contributions

KJ, AR, and KB designed the study. KJ, MN, and ML performed the experiments. KB, AR, KJ, and ML analyzed the data. AR, KB, and KJ wrote the manuscript. KJ and MN designed and prepared the figures. KB provided financial support.

### Conflict of interest statement

The authors declare that the research was conducted in the absence of any commercial or financial relationships that could be construed as a potential conflict of interest.
