# Supplementary material for: OmpR-Mediated Transcriptional Regulation and Function of Two Heme Receptor Proteins of Yersinia enterocolitica Bio-Serotype 2/O:9
Source: Front Cell Infect Microbiol. 2018 Sep 20;8:333. doi: 10.3389/fcimb.2018.00333 (PMC6158557; doi:10.3389/fcimb.2018.00333)
Supplement: Supplementary file 1 [file Data_Sheet_1.docx]

*hem*-1 locus

**ATG**GATAAACAGTTGAACAAAGCACCCACAATGAATGACGAGCCTGCAGCCAAACCTCCTGCGGGCAACAAGCCCCTGTCTGTCTCCAGCGAGCAATTGCTGGGAGAGCATAGTGTCGCTTTTATCATCCATCAGGGCGAATGCTATCAACTGCGCCAGACCAAAGCAGGGAAACTGATACTGACTAAA**TAA**TAGCCCAATGCCAATGTCGTGACAGCAAGGTAGCGGTTCCCGCTAGCACCGCTGTGGCAGGGGCAAAGGATACATCGCAAGCCACCCAGATTTTAGAATCAAGGCAGCCAGCAACCTATTTATTTGTTTTGCATATGATTTTTTTGCATAGAAAATATGGAGAATTGCCGAC**ATG**CCGCGTTCCACTTCCGACCGTTTCCGTTGGTCCCCACTCAGTTTGGCAATCGCTTGCACTTTGCCACTTGCTGTTCAAGCTGCTGATACCTCGTCCACTCAAACCAACAGCAAAAAACGTACTGCCGATACCATGGTAGTAACTGCGACCGGTAATGAGCGCAGCAGTTTTGAAGCACCGATGATGGTGACAGTGGTTGAGGCCGATACACCGACCAGCGAAACCGCCACCTCTGCCACCGATATGCTGCGCAATATTCCAGGCCTGACCGTCACTGGCAGTGGGCGCGTTAACGGGCAGGACGTGACACTGCGTGGCTACGGCAAACAAGGTGTGCTGACTTTGGTTGATGGTATTCGCCAAGGCACTGACACCGGCCACCTGAACTCTACCTTCCTCGATCCGGCGCTGGTTAAGCGTGTTGAAATCGTCCGCGGCCCATCAGCGTTGCTGTATGGTAGCGGTGCCTTGGGCGGGGTTATTTCTTATGAAACCGTTGATGCCACCGATCTCTTATTACCGGGCCAAAATAGCGGCTATCGGGTTTACAGCGCTGCGGCGACCGGTGATCACAGCTTCGGCTTGGGTGCCAGTGCTTTTGGCCGCACCGATGATGTCGATGGCATTCTCTCTTTTGGTACTCGTGATATCGGCAATATTCGCCAAAGCGACGGTTTTAACGCACCAAATGACGAAACCATCAGTAATGTGCTGGCAAAAGGCACCTGGCGTATTGACCAGATTCAGTCGTTAAGTGCCAATCTGCGCTATTACAACAACAGCGCACTGGAGCCAAAGAATCCGCAAACCAGTGCGGCATCCAGCACCAATCTTATGACCGACCGCTCGACTATCCAACGTGATGCACAGCTTAAATACAACATTAAGCCACTTGATCAAGAATGGTTGAATGCCACCGCGCAAGTTTACTACTCCGAAGTGGAAATCAATGCACGACCACAAGGCACACCAGAAGAGGGACGCAAGCAAACCACCAAAGGTGGCAAGCTGGAAAACCGCACTCGTCTGTTCACCGACAGTTTTGCATCACACTTGCTGACTTACGGTACAGAAGCCTATAAACAGGAACAAACACCGGGCGGCGCAACAGAAAGTTTCCCTCAGGCAGATATCCGCTTTGGTTCTGGCTGGCTGCAAGATGAAATCACCTTACGCGACCTGCCGGTTTCTATTTTGGCTGGAACCCGTTATGACAACTATCGCGGCAGCAGCGAAGGCTATGCCGATGTGGATGCCGATAAATGGTCATCTCGTGGTGCCGTCAGTGTGACACCGACAGACTGGCTGATGCTATTTGGTTCCTATGCTCAGGCTTTCCGTGCTCCGACCATGGGCGAGATGTACAACGATTCGAAACACTTTTCGATGAATATCATGGGTAACACCCTGACCAACTATTGGGTACCCAACCCGAATCTGAAACCGGAAACCAACGAAACTCAAGAGTACGGTTTTGGCCTGCGCTTTAACGACCTGATGATGGCTGAGGATGATCTGCAATTCAAAGCCAGCTACTTTGATACCAACGCCAAAGACTATATCTCCACCGGCGTTACGATGGACTTCGGCTTCGGGCCAGGTGGCTTGTACTGCAAAAACTGCTCGACCTATTCCACCAATATTGATCGGGCAAAAATCTGGGGTTGGGATGCCACTATGACTTACCAGACTCAGTGGTTTAACTTGGGTCTGGCCTATAACCGCACCCGTGGTAAAAACCAAAATACCAATGAATGGCTCGATACCATTAACCCGGATACCGTTACCAGTACCCTGGATGTACCCGTTGCTAACTCCGGCTTTGCTGTGGGTTGGATCGGAACATTTGCTGATCGCTCTAGCCGAGTCTCCAGCTCAGGCACACCGCAAGCCGGTTATGGCGTCAATGACTTCTACGTCAGCTATAAAGGCCAAGAGCAATTTAAAGGTATGACCACCACCGTGGTGTTGGGTAACGCATTCGATAAAGAGTATTACGCGCCACAAGGCGTGCCACAGGATGGTCGTAACGCGAAGTTCTTCGTGAGCTATCAGTGG**TAA**CTGAATACAAAAGTTAGTCTGAAATAACACTTCCGGTAATTCAATCAGCGAATTACCGGATTATGATTTCTACCTGTCACCTGCATATAAAAAATCAATAGAACGAGGAAGTTACT**ATG**AGCAAATCAATATACGAGCAGTATCTACAAGCTAAAGCAGATAATCCGGGTAAATATGCGCGTGATTTGGCCACTCTGATGGGGATTTCAGAAGCGGAACTGACCCATAGCCGCGTTGGTCATGATGCCAAACGTCTGAAAGGTGATGCCCGCGCACTACTGGCCGCATTGGAAGCTGTCGGTGAGGTCAAAGCTATCACCCGCAACACCTATGCTGTACATGAGCAAATGGGCCGTTACGAAAATCAACATCTGAATGGTCATGCTGGTTTGATCCTCAATCCACGCAATTTAGATTTACGCCTGTTCCTCAACCAGTGGGCCAGTGCATTCACGCTGACAGAAGAAACTCGCCACGGTGTACGCCATAGCATCCAGTTTTTCGACCATCAAGGCGATGCTCTGCATAAAGTGTATGTCACTGAACAAACTGATATGCCAGCCTGGGAAGCGCTACTGGCGCAGTTTATCACCACAGAAAATCCAGAGTTGCAGCCAGAGCCACTGAGCGCACCTGAAGTCACTGAACCGACAGCCACCGATGAAGCTGTAGATGCTGAATGGCGTGCTATGACTGACGTGCATCAGTTCTTCCAGTTGCTCAAACGCAATAATCTGACCCGTCAGCAAGCCTTCCGTGCCGTGGGTAATGATCTGGCTTATCAGGTTGATAACAGTTCTCTGACCCAGTTGCTGAACATTGCTCAGCAAGAACAGAATGAAATCATGATTTTTGTGGGTAACCGTGGCTGTGTACAAATATTCACCGGCATGATTGAAAAGGTTACACCACATCAAGATTGGATTAATGTTTTCAACAAGCGCTTCACGCTGCATCTGATTGAAACAACGATTGCTGAAAGCTGGATTACCCGCAAGCCAACAAAAGACGGTTTCGTGACCAGTTTGGAACTGTTTGCTGCTGATGGCACCCAAATTGCACAACTTTACGGTCAGCGCACCGAAGGCCAGCCAGAACAAACGCAATGGCGTGAGCAAATTGCTCGCCTCAATAATAAGGATATCGCCGC**ATGA**GACTAAGGTTACTGTCACTCCCTTTCATTCTGTCGCTGAGCGCCTGTCTTCTGCCGCTGAACTCTTTCGCCGCAGAACGTATCGTCACCATTGGCGGTGATGTCACAGAAATCGCCTACGCACTGGGTGCAGGTGGTGAGATTGTGGCTCGTGACAGTACCAGTCTACAGCCTCAGGCTGTGCAAAAGCTGCCTGATGTTGGTTACATGCGCATGCTTAATGCCGAAGGGATTTTGGCAATGAAGCCAACCATGCTGCTGGTCAGTGAGCTGGCGCAACCTTCACTGGTACTCAAACAAGTCGCGGACAGTGGTGTGAATGTTGTCACTGTACCGGGTCAAACCACACCTGAAAGTGTGGCAGTGAAGATAAATGCTGTTGCCAGCGCACTGCATCAGCAGGAAAAAGGGCAAGCGCTTATCAAAGACTATCAGCAACGTTTAGCCGCAGTGAACAACACACCACTGCCAGTTAAGGTGCTGTTTGTCATGAGTCATGGCGGCTTAACCCCGATGGCAGCCGGTCAAAATACCGCAGCGGACGCCATGATCCGCGCCGCCGGTGGTAGAAATGCGATGCAAGGTTTTAGCCGCTATCGTCCGTTATCACAGGAAGGAGTGATTGCCAGTGCGCCGGATTTATTACTGATCACCAGTGACGGTGTGAGAGCGCTGGGTGGCAGTGAAGGTATCTGGAAATTACCGGGGATGGCATTGACCCCGGCGGGCAAAAATAAACGCCTGTTAGTGGTTGATGATATGGCGCTACTCGGCTTTGGCTTGGAAACACCGCAAGTGCTATCGCAACTGCGCAAAGGTATGGAACAAGCGCA**ATGA**ATTGCCGTATTCACCCGCGATTCATGCTGAGTATCCTGCTGATGATCCTGATCATCCTGGCACTCGGTTCGGCCAATATGGGTGCATTGACGCTCTCGTTTCGTACCTTGTGGCATGCATCATTAGATGATGCCATGTGGCATATTTGGCTAAATATCCGCCTGCCCCGCGTACTGTTGGCGGTAGTGGTCGGCTGCGCACTGGCAGTTTCCGGCGCGATCATGCAGGGGTTATTCCGCAACCCACTGGCAGACCCCGGTCTGTTGGGGATCAGCAGTGGCGCAGCCCTGTGTGTCGGGCTGATTATTGTGATGCCATTCAGTTTGCCGCCACTGCTGGCGCTTTACAGTCATATGGTCGGTGCCTTTATTGGCAGTCTGGCCATTTCTGCCATTATTTTCACCCTCAGCCGCTGGGGGCATGGCAACTTATCCCGTTTGCTGCTGGCCGGCATTGCGATCAATGCGCTATGTGGTGCAGCGGTCGGTGTGCTGACTTATATCAGTGATGATCAACAATTACGTCAGTTCTCCCTGTGGAGCATGGGCAGTTTAGGTCAGGCGCAGTGGTCGACACTCATGGTGGCGGCATCATTGATTCTGCCTGCGTGCGTGCTCGGTTTGCTGCAAGCACGTCAGTTGAACCTGTTGCAGCTAGGAGATGAAGAAGCGCACTACCTCGGCGTCAATGTAAAGCAAGCCAAACTGCGCTTGCTGTTACTCAGCGCTATTTTGATTGGTGCCGCCGTCGCTGTCAGTGGCGTTATTGGTTTTATCGGGTTAGTGGTACCGCATCTTATCCGGATGCGGATCGGGGCTGACCATCGCTGGTTACTACCCGGCGCTGCGCTGGGTGGAGCTTGCCTGTTACTGACCGCCGATACACTAGCTCGAACTCTGGTCGCACCGGCAGAAATGCCGGTCGGGTTAATCACCAGCCTACTGGGTGGCCCTTATTTTCTGTGGCTGATTTTACGTCAGCGGGAGCAACGCA**GTG**GT**TGA**TACGGCGCTATTAGAAGCGAATCAGCTTTCCTACCATGTACAGGGGCAAAAGCTGATTAATAACGTTTCGCTACAAATTACCAGCGGCGAAATGGTAGCGATTATCGGGCCAAACGGTGCGGGGAAATCCACTCTGTTACGTTTATTAACCGGCTATCTCGCACCATCTGAAGGTCACTGCCAGTTACTGGGTAAGAACCTCAATAGCTGGCAGCCCCAAGCATTAGCCCGAACCCGAGCGGTGATGCGTCAATACAGCGATTTGGCCTTTCCATTCAGTGTTAGCGAAGTGATTCAGATGGGCCGAGCACCTTATGGGGCCGCGCAAAATCGTCAGGCGTTGCAAGAAGTGATGGCGCAAACCGACTGTCTGGCACTGGCGCAGCGGGATTACCGCGCACTGTCCGGAGGCGAGCAACAGCGGGTTCAACTCGCTCGTGTGTTAGCGCAACTGTGGCAACCGGAACCGACCCCACGCTGGTTGTTCCTCGACGAACCAACCTCGGCATTGGATTTGTATCATCAACAGCATACCTTGCGCTTATTACGCCAGCTCACCCTTCAGGAACCCCTGGCGGTGTGCTGTGTGCTGCATGATTTGAATCTGGCCGCACTCTATGCCGATCGTATTTTGCTGTTAGCACAGGGCGAACTGGTGGCCTGTGGTACACCAGAGGAAGTGCTCAATGCCGAAACACTAACCCGCTGGTATCAGGCTGATTTGGGCATTTCGCGTCACCCTGAAAGCGCCCTGCCACAGATCTATTTGCGTCAG**TAA**

*hem*-2 locus

**ATG**CACAAAGCAACATCCATAACGCAAAATGCAACCGACCAAACCGTTTCCTCTTTGCCACTTTCATGTATTAACAGCCAACAATTGCTTGGACAACATGAAGTTGTCGCTATCAATCACCAGGGTCAACTTTATTACTTACGCCAGACCAAAGCGGGAAAACTGATACTGACGAAA**TAA**CCTTCAGTCACGCCCCTTGCTTAACACCATTGCCCCGACGCCAGCCACCGCAGGTTTGATACCAAGGCAGCCAGCAATCCATTGATTATATTAAACAATATATGGAGAGCTTTTA**ATG**CCTCATTTCTTTCGATTATCTTCATGCAGTCTGGCTATCAGCTTGGCACTTTCGTCAGTGACGGCCGCCAACCCACCGGAAAAAAAGCACCATGACACGCTGAAAGTGACAGGAACTCGCGCCGTGACTGACAGCTTTCAGTATCCCGGCACCATGACAGTTATTGATGCCTCCTCGCCACAAAATCAAACCGCCACCAGCGCCGCTGAAATGTTAAAAAATATACCCGGGGTGAGTGTCACCGGGGTTGGCCGCACCAATGGGCAGACCGTCAATATCCGAGGTTATGACCAATACGGCGTACTGGTGTTAATTGATGGCATTCGTCAGGGAATTAATGGCGCACATTTTAATGGCACTTTCCTTGATCCGGCATTAATCAAACAAGTCAGTGTGATACGCAGCCCGTCAACCGCGCTATTTGGCAGTGGTGCTTTAGGGGGCGTTATTGCTTACGAGACTGTTAATGCAGCTGATTTACTGGCTGATGATGAAAACTTTGGTATGCGAATTGGCCATTATAGCGCCAGTGCATACCACAGTCAGGGCCTTGCGATGTCGGCTTTTGGCCGAAGCGAAAACCTCGACGGCATTATTGCCTTGAGTAAACGCAAGGTGGGAAATCTTCGCCAAAGTAATGGTTACGACGCCCCCAACAAGGAAGCGATTAATAGCCTGATGCTGAAAGGCACCCTCAACCTCTCTGATAACCAGTCATTAACCACCGCCCTGCGTTATTACAATAACCGGGCGCGGCAACCGCGTATGGCTCATCAGAGTGCGCCTAATTTAGAAAAGAATATAAGCCCGATGATAAATCGCTCGACTATTCAAAGAGATGCCGAACTCACTTGGCATTTGCAACCCGAACACCTTGATTGGCTGGATGCCACCACCCAGATTTACACTACTGAAATTAATGTCAATGATGATGTCCCTGTTAAAGGAGAGGGATATGGCAGCCGCAAGCAGGTAACGCGCGGAATTAAACTGGAGAACCGCAGTAAGCTCTTTACCGACAGCCCGGCCGCGCATCTCTTGACCTACGGTATTGAAACCAATCACCAGCGGCAAATCCCTAAAGGGGCTATTCGCAGCTTCCCGCCTGCTGAAATCAAATTGTCCTCCGGTTGGCTACAAAATGAAGTGACTTTGCGCGACTTACCCGTCACTTTGTTGGCCAGCACCCGTTTCGACAGCTACAAAAATAGCCGCGAGGGTTTTGCCGATAAAGAGGCAAAAAACCAGTCAACACGCGGGGCAGTGACGGTCAACCCAACTGACTGGTTAATGCTATTTGGTGCTTACAGCCAGGCATTTCGCACCCCGACATTGACTGAGCTTTATAATAATTCACTCCATTTCGATATGTGGATTGCTAAAAACTACTGGCAGCCCAATCCAAACCTAAAACCAGAAAGTAATGTCACCCGTGAAGCCGGTTTTGGTCTGCATTTTGATAATCTGCTGGCTGATGACGATGCTGTTAAATTGAAAGCCAGTTATTTTCATATTGATGCCAAAGACCGCATTACTAGTGAAGTCCACGCGAACCCTTTTGGTAAGAATTACTCCGGTTACATTAATATCCCCCGCAGCAAGTCCTGGGGCTGGGATGCTTCGCTGGATTATCAAAACCGCTGGTTTGACTGGAATCTGGCCTATAACCGCACCCGCGGTATTAATCTCGATACCCGCCAATTTATTAACAGCATCAACCCTGACACCGTCACCAGCCGCCTGAATATTCCCATTGCAAACAGCGGATTTAGCACCGGCTGGGTAGCCACCATGGCGGAAAACACCAAATTCATGAAAAATAATGACGCCAAAACATCCAAAAAAGCACCTTATAAACCGCAAGCGGGTTATACCGTACATGACTTTTATCTGAGTTATCGCGGGCAAGGCGCACTCAAAGGTGTCACCACCACGGCGGTACTGGGTAATGCCTTTAATAAAAAACATTACTCGCCACAAGAAATTCCTCGTGATGGGCGCAACGCTAAGGTGCTGATCAGTTATCAGTGG**TGA**TATTTCATCTGCCTGATGGTGGTAGGTTAAAAAACAATCATAATAAAATGGCTCTATGGAACCCTGCCATCGCACACCCGCTATGGCACTATATTATAAAGGAATCAATCA**ATG**GAAATGACACTGTCTCAACGCTACCTCAACACCAAGCAAACTCGCCCAGAACTTTCCTCGCGTGATTTGGCTCAAAAACTGAATATCAGTGAAGCGGAACTGACCTATGCCCGTGTCGGTGATGATGCTGAACGATTGGATATCAGCGCCAGTGTATTGCTTGCCGAGTTAGAACATGTTGGCGTCACTTGCTCGGTGACGTCCAACCCACATGCAGTACATCAGATAATGGGAGAGTATCAAAACTTACGGCTGCATGGACATTTGGGTTTAATCCTCAACCCGCGCACCCTGGATTTAAGGCTATTTTTTCGCCACTGGAATGCTGTTTTCAGTTTGCGCGAAACCACAGCGCAGGGTGAACAACTCAGCATTCAATGTTTTGATTTCCAAGGCAATGCCATTCATCAAATCTATTGTACCGAGAAAACAAACCAAGAGGCCTGGCAAGCATTGGTCGCCAAGTATCGTACGGCGAATAATTCTCCGCTAACAATAGAACCTGCCAATGAGGCCCCCACCACTCAATCTTCCATCGACAACACAATTATTGATGCTGAATGGCGGAAACTGACTGATATTCATCAATTTTTTATGTTACTGAAACGCCATAACATTAGCCGACAACAAGCCTTCCGCGCGGTTGGCGATGATCTGGCGTATCAAGTCGATAATCAAGCTGTAATACAAATACTTAAGGCGGCACAAGCGGATCTGAATGAAATTATGTTGTTCGTCGGTAACAGTGGATGCATGCAGATATTCACCGGTGCTATTGAACAATTATCAGCCCTGGAAGAAAATCAATCAGCTGATGGTCAGTGGGTTAACGTAAGCAATCCTCGATTTGATTTACAGCTAAACCAACAGGCTATTACAGAAAGTTGGGTGACACGTAAACCGACCAAAGATGGCTTTGTCTCAAGCCTGGAGTTGCTCGATGAGCATGGGAAGCATATCTTACAAATTTTTGGTCAACGCAGCGAAGGCCAGCCAGAGCAAAATCAATGGCATCAACAACTGGCTGAATTGCCCCCAATAGGCGTGTCGC**ATG**AA**TAA**ATGGCGCGCGGCACTTAGATTTATCATTTCACTGTCATTTGTTATTTCAATGAATTGTATTGCCACACCACGTATTGTGACCCTTGGCGGCGATGTCTCTGAAATCACTTATGCACTCGATGCCGGTGACCTTATCGTGGGCCGGGATAGCACCAGCCTGACTCCTGATGCATTGAAAGCGCTACCCGATGTGGGTTATATGCGCCTGCTCAATGCTGAAGGTATTCTTGCTTTAAAACCCACCTTGATTTTGAGCAGTGAGCGCGCCGGGCCATCAAGAGTACTGAAACAAGTGATGGAGTATGGAGTCAAGCTTATCTATGTCCCCGCCGATAAATCGCCGCAAGGCGTGATAGATAAGATACAGTTGATTGCCACCACCGTCAGTCAAGAAGAGAAAGGCCGGCAGTTGATACAACATTATCAACAACAGTTGGACACTGTGGTATCAAGCCCGCTACCGGTGAAAGCATTGTTTGTCATGATTCATGCCGGAATTCCTCCGTTGGCCGCGGGTTTAGATACCGCAGCTGATAGCATGTTCAAGGCATCGGGGCTTAAAAATGCGATTAAGGAATTCAGCGGTTATCGTCCGCTATCACAAGAAGGGATTATCGACAGTGCCCCTGATTTACTCATCGTGACCACACATGGTGTGGCATCACTCAAGGGGGTAGAGAATGTCTGGCGGCTGCCGGGGCTTGCTCTCACACCCGCGGGGAAACAAAAACGGCTGCTAGTACTTGATGATATTGCCTTGTTGGGGTTTGGTTTACAGACCCCGGATGTTCTTAAGCAACTGCGTGCGGCGGCTGAATCAAAC**TGA**

**Figure S1 ǀ** **Sequences of the *hem*-1 and *hem*-2 loci*.*** START and STOP codons are in bold.
