# Supplementary material for: OmpR-Mediated Transcriptional Regulation and Function of Two Heme Receptor Proteins of Yersinia enterocolitica Bio-Serotype 2/O:9
Source: Front Cell Infect Microbiol. 2018 Sep 20;8:333. doi: 10.3389/fcimb.2018.00333 (PMC6158557; doi:10.3389/fcimb.2018.00333)
Supplement: Supplementary file 2 [file Data_Sheet_2.docx]

(A)

HemP1 23 LNKAPTMNDEPAAKPPAGNKPLS-VSSEQLLGEHSVAFIIHQGECYQLRQTKAGKLILTK 81

++KA ++ + A + PLS ++S+QLLG+H V I HQG+ Y LRQTKAGKLILTK

HemP2 1 MHKATSIT-QNATDQTVSSLPLSCINSQQLLGQHEVVAINHQGQLYYLRQTKAGKLILTK 59

Sequence identity: 52%

(B)

HemS1 1 MSKSIYEQYLQAKADNPGKYARDLATLMGISEAELTHSRVGHDAKRLKGDARALLAALEA 60

M ++ ++YL K P +RDLA + ISEAELT++RVG DA+RL A LLA LE

HemS2 1 MEMTLSQRYLNTKQTRPELSSRDLAQKLNISEAELTYARVGDDAERLDISASVLLAELEH 60

HemS1 61 VGEVKAITRNTYAVHEQMGRYENQHLNGHAGLILNPRNLDLRLFLNQWASAFTLTEETRH 120

VG ++T N +AVH+ MG Y+N L+GH GLILNPR LDLRLF W + F+L E T

HemS2 61 VGVTCSVTSNPHAVHQIMGEYQNLRLHGHLGLILNPRTLDLRLFFRHWNAVFSLRETTAQ 120

HemS1 121 GVRHSIQFFDHQGDALHKVYVTEQTDMPAWEALLAQFITTENPELQPEPLSAPEVTEPTA 180

G + SIQ FD QG+A+H++Y TE+T+ AW+AL+A++ T N L EP + T+ +

HemS2 121 GEQLSIQCFDFQGNAIHQIYCTEKTNQEAWQALVAKYRTANNSPLTIEPANEAPTTQSSI 180

HemS1 181 TDEAVDAEWRAMTDVHQFFQLLKRNNLTRQQAFRAVGNDLAYQVDNSSLTQLLNIAQQEQ 240

+ +DAEWR +TD+HQFF LLKR+N++RQQAFRAVG+DLAYQVDN ++ Q+L AQ +

HemS2 181 DNTIIDAEWRKLTDIHQFFMLLKRHNISRQQAFRAVGDDLAYQVDNQAVIQILKAAQADL 240

HemS1 241 NEIMIFVGNRGCVQIFTGMIEKVTPHQD-------WINVFNKRFTLHLIETTIAESWITR 293

NEIM+FVGN GC+QIFTG IE+++ ++ W+NV N RF L L + I ESW+TR

HemS2 241 NEIMLFVGNSGCMQIFTGAIEQLSALEENQSADGQWVNVSNPRFDLQLNQQAITESWVTR 300

HemS1 294 KPTKDGFVTSLELFAADGTQIAQLYGQRTEGQPEQTQWREQIARL 338

KPTKDGFV+SLEL G I Q++GQR+EGQPEQ QW +Q+A L

HemS2 301 KPTKDGFVSSLELLDEHGKHILQIFGQRSEGQPEQNQWHQQLAEL 345

Sequence identity: 53%

(C)

HemT1 7 SLPFILSLSACLLPLNSFAAERIVTIGGDVTEIAYALGAGGEIVARDSTSLQPQAVQKLP 66

+L FI+SLS ++ +N A RIVT+GGDV+EI YAL AG IV RDSTSL P A++ LP

HemT2 7 ALRFIISLS-FVISMNCIATPRIVTLGGDVSEITYALDAGDLIVGRDSTSLTPDALKALP 65

HemT1 67 DVGYMRMLNAEGILAMKPTMLLVSELAQPSLVLKQVADSGVNVVTVPGQTTPESVAVKIN 126

DVGYMR+LNAEGILA+KPT++L SE A PS VLKQV + GV ++ VP +P+ V KI

HemT2 66 DVGYMRLLNAEGILALKPTLILSSERAGPSRVLKQVMEYGVKLIYVPADKSPQGVIDKIQ 125

HemT1 127 AVASALHQQEKGQALIKDYQQRLAAVNNTPLPVKVLFVMSHGGLTPMAAGQNTAADAMIR 186

+A+ + Q+EKG+ LI+ YQQ+L V ++PLPVK LFVM H G+ P+AAG +TAAD+M +

HemT2 126 LIATTVSQEEKGRQLIQHYQQQLDTVVSSPLPVKALFVMIHAGIPPLAAGLDTAADSMFK 185

HemT1 187 AAGGRNAMQGFSRYRPLSQEGVIASAPDLLLITSDGVRALGGSEGIWKLPGMALTPAGKN 246

A+G +NA++ FS YRPLSQEG+I SAPDLL++T+ GV +L G E +W+LPG+ALTPAGK

HemT2 186 ASGLKNAIKEFSGYRPLSQEGIIDSAPDLLIVTTHGVASLKGVENVWRLPGLALTPAGKQ 245

HemT1 247 KRLLVVDDMALLGFGLETPQVLSQLRKGME 276

KRLLV+DD+ALLGFGL+TP VL QLR E

HemT2 246 KRLLVLDDIALLGFGLQTPDVLKQLRAAAE 275

Sequence identity: 60%

**Figure S2 ǀ** **Protein sequence alignments of homologous HemP, HemS and HemT proteins encoded within the *hem*-1 and *hem*-2 loci*.*** Alignments obtained using Blastp (https://blast.ncbi.nlm.nih.gov/ Blast.cgi).
