# Supplementary material for: OmpR-Mediated Transcriptional Regulation and Function of Two Heme Receptor Proteins of Yersinia enterocolitica Bio-Serotype 2/O:9
Source: Front Cell Infect Microbiol. 2018 Sep 20;8:333. doi: 10.3389/fcimb.2018.00333 (PMC6158557; doi:10.3389/fcimb.2018.00333)
Supplement: Supplementary file 3 [file Table_1.DOCX]

**Table S1. Strains and plasmids used in this study**.

| **Strains and plasmids** | **Description** | **Reference or source** |
| --- | --- | --- |
| ***Y. enterocolitica* O:9** | | |
| Ye9 | clinical isolate of serotype O:9, carrying virulence plasmid pYV | Clinical isolate, laboratory collection |
| Ye9N | Ye9 derivative, spontaneous Nal^R^ mutant | Brzostek et al., 2007 |
| AR4 | Ye9N derivative Δ*ompR*::Km defective in OmpR production, Nal^R^, Km^R^ | Brzostek et al., 2003 |
| Ye9*fur* | Ye9N derivative Δ*fur*::Gm defective in Fur production, Nal^R^, Gm^R^ | This work |
| AR4*fur* | AR4 derivative Δ*fur*::Gm defective in Fur and OmpR production, Nal^R^, Km^R^, Gm^R^ | This work |
| ***Y. enterocolitica* O:8** | | |
| JB580v | 8081 derivative, R^-^ M^+^, pYV^+^ | Kinder et al. 1993 |
| ***E. coli*** | | |
| S17-1 λ*pir* | Tp^r^, Str^r^, *pro*, *thi*, *recA*, *hsdR514*, (r^-^ m^+^), *λpir*, RP4: 2- Tc::Mu-Km^r^::Tn7 | Simon et al., 1983 |
| DH5α | F^–^, *endA1*, *glnV44*, *thi-1*, *recA1*, *relA1*, *gyrA96*, *deoR*, *nupG*, *purB20*, φ80*lacZ*ΔM15, Δ(*lacZYA-argF*)U169, hsdR17(*rK*–*mK*+), λ^–^ | Sambrook et al., 1989 |
| BL21 (DE3) | F^-^, *ompT hsdSB* (r_B-_m_B-_) *gal*, *dcm* (DE3) | Life Technologies |
| SASX77 | *lacY1*, *glnX44*(AS), *hemA8*, *rpsL134*(strR), *malT1*(λ^R^), *metB1* | Sasarman et al., 1968, CGSC Collection |
| BW25113 | F^-^,*Δ(araD-araB)567*, *ΔlacZ4787*(::rrnB-3), *λ^-^*, *rph-1*, *Δ(rhaD-rhaB)568*, *hsdR514* | Baba et al., 2006  Keio Collection |
| PLASMIDS | | |
| pFUSE | suicide vector, derivative of pEP185.2 with promoterless *lacZYA* genes, Cm^R^ | Baumler et al., 1996 |
| pDS132 | *ori* R6K (narrow host range, replication only in *E. coli* λ*pir*), *oriT* RK2, *sacB*, Cm^R^ | Philippe  et al., 2004 |
| pBBR1MCS-5 | broad-host-range cloning vector, *ori* pBBR1, Mob^+^, *oriT* RK2, Gm^R^ | Kovach et al*.*, 1995 |
| pDSfur | pDS132 derivative carrying 1860 bp cassette for *fur* mutagenesis constructed by overlap extension PCR cloned between XbaI sites of the vector, Gm^R^ | This study |
| pCM132Gm | pCM132 derivative, ori pMB1, ori RK2, oriT RK2, promoterless *lacZ* gene, Gm^R^ | DBG collection** |
| pCM1 | pCM132Gm derivative carrying 277 bp upstream of *hemP1* start codon and 37 bp of ORF *hemP1* cloned upstream of promoterless *lacZ* gene between EcoRI and KpnI sites | This study |
| pCM2 | pCM132Gm derivative carrying 175 bp upstream of *hemP2* start codon and 56 bp of ORF *hemP2* cloned upstream of promoterless *lacZ* gene between EcoRI and KpnI sites | This study |
| pFX-P | Golden Gate-compatible pDSK602 derivative without promoter for generation of translational mRNA::*gfp* fusions; Sp^R^ | Schmidtke et al., 2013 |
| pFX-0 | Promoterless pFX-P derivative; control plasmid for GFP reporter fusions; Sp^R^ | Schmidtke et al., 2013 |
| pFX-1 | pFX-P derivative carrying *gfp* under control of p_lac_ promoter | Schmidtke et al., 2013 |
| pFX-P_lac_-hemR | pFX-P derivative carrying untranslated region of *hemR* (5'UTR) with the first 16 codons of ORF *hemR*, and *lacZ* promoter | This study |
| pETOmpR | pET28a carrying the entire *ompR* coding sequence (725 bp fragment), Km^R^ | Nieckarz et al., 2016 |
| pBOmpR | pBBR1MCS-3 with XhoI/PstI fragment containing entire coding sequence of *ompR* (ORF with rbs), Tet^R^, previously named as pBR3 | Brzostek et al., 2007 |
| pBBR1 MCS-3 | broad-host-range cloning vector, *ori* pBBR1, Mob^+^, *oriT* RK2, Tet^R^ | Kovach et al*.*, 1995 |
| pRK2013 | helper plasmid used to mobilize vectors in triparental mating, Km^R^ | Ditta et al., 1980 |
| pACYC184 | *ori*p15A, medium copy number, Cm^R^, Tc^R^ | Chang and Cohen, 1978 |
| pHEM1 | pACYC184 with *hemPR1* with its own promoter (2634 bp) amplified with primers hemPR-F and hemPR-R and cloned within EcoRI site disrupting Cm casette; Tc^R^. | This study |
| pHEM2 | pACYC184 with *hemPR2* with its own promoter (2540 bp) amplified with primers hemPR2-F and hemPR2-R and cloned within EcoRI site disrupting Cm casette; Tc^R^. | This study |
| *PCM Collection, Polish Collection of Microorganisms - Ludwik Hirszfeld Institute of Immunology and Experimental Therapy, Polish Academy of Sciences (ECCO) | | |
| **Department of Bacterial Genetics*,* Institute of Microbiology, Faculty of Biology, University of Warsaw | | |
| Cm^R^ chloramphenicol resistance, Gm^R^ gentamicin resistance, Km^R^ kanamycin resistance, Nal^R^ nalidixic acid resistance, Sm^R^ streptomycin resistance, Tet^R^ tetracycline resistance, ::Km, insertion of kanamycin resistance cassette; ::Gm, insertion of gentamicin resistance cassette | | |

**REFERENCES**

Ditta, G., Stanfield, S., Corbin, D., Helinski, D.R. (1980). Broad host range DNA cloning system for gram-negative bacteria: construction of a gene bank of Rhizobium meliloti. *Proc. Natl. Acad.* *Sci.* U.S.A. 77, 7347–7351.

Brzostek, K., Raczkowska, A., and Zasada, A. (2003). The osmotic regulator OmpR is involved in the response of *Yersinia enterocolitica* O:9 to environmental stresses and survival within macrophages. *FEMS Microbiol Lett*. 228, 265–271.

Brzostek, K., Brzóstkowska, M., Bukowska, I., Karwicka, E., and Raczkowska, A. (2007). OmpR negatively regulates expression of invasin in *Yersinia enterocolitica. Microbiol*. 153, 2416–2425. doi: 10.1099/mic.0.2006/003202-0

Kinder, S.A., Badger, J.L., Bryant, G.O., Pepe, J.C., Miller, V.L. (1993) Cloning of the *Yen*I restriction endonuclease and methyltransferase from *Yersinia enterocolitica* serotype O8 and construction of a transformable R^−^M^+^ mutant. Gene. 136, 271-275. doi: 10.1016/0378-1119(93)90478-L

Kovach, M.E., Elzer, P.H., Hill, D.S., Robertson, G.T., Farris, M.A., Roop, R.M., and Peterson, K.M. (1995). Four new derivatives of the broad-host-range cloning vector pBBR1MCS, carrying different antibiotic-resistance cassettes. *Gene*. 166, 175–176.

Nieckarz, M., Raczkowska, A., Dębski, J., Kistowski, M., Dadlez, M., Heesemann, J., et al. (2016). Impact of OmpR on the membrane proteome of Yersinia enterocolitica in different environments: repression of major adhesin YadA and heme receptor HemR. *Environ*. *Microbiol*. 18, 997–1021, doi: 10.1111/1462-2920.13165.

Philippe, N., Alcaraz, J.P., Coursange, E., Geiselmann, J., and Schneider. D. (2004). Improvement of pCVD442, a suicide plasmid for gene allele exchange in bacteria. *Plasmid.* 51, 246–255. doi: 10.1016/j.plasmid.2004.02.003

Sambrook, J., Fritsch, E.F., and Maniatis, T. (1989). Molecular Cloning: a Laboratory Manual. 2nd ed. Cold Spring Habor, NY, USA: Cold Spring Harbor Laboratory Press.

Simon, R., Priefer, U., and Pühler, A. (1983). A broad host range mobilization system for *in vivo* genetic engineering: transposon mutagenesis in Gram negative bacteria. *Nat. Biotechnol.* 1, 784–791.

Chang AC, and Cohen SN. (1978). Construction and characterization of amplifiable multicopy DNA cloning vehicles derived from the P15A cryptic miniplasmid. *J Bacteriol.* 134(3):1141-56.
