# Supplementary material for: OmpR-Mediated Transcriptional Regulation and Function of Two Heme Receptor Proteins of Yersinia enterocolitica Bio-Serotype 2/O:9
Source: Front Cell Infect Microbiol. 2018 Sep 20;8:333. doi: 10.3389/fcimb.2018.00333 (PMC6158557; doi:10.3389/fcimb.2018.00333)
Supplement: Supplementary file 4 [file Table_2.DOCX]

**Table S2. Oligonucleotide primers used in this study.**

| **Purpose and Target** | **Name of primer** | **Primer sequence (5’→ 3’)*** | **Restriction enzyme** | **Reference** |
| --- | --- | --- | --- | --- |
| sqRT-PCR *hemPRSTUV*-1 | RThPR1F | CACCCACAATGAATGACGAG |  | This study |
|  | RThPR1R | AAGGCACCGCTACCATACAG |  | This study |
|  | RThRS1F | GTTGGATCGGAACATTTGCT |  | This study |
|  | RThRS1R | ATGGTCGAAAAACTGGATGC |  | This study |
|  | RThPV1R | GCTGCCAGCTATTGAGGTTC |  | This study |
| sqRT-PCR  *hemPRST*-2 | RThPR2F | CCAAAGCGGGAAAACTGATA |  | This study |
|  | RThPR2R | TAATGCCGGATCAAGGAAAG |  | This study |
|  | RThRS2F | TAATCTGCTGGCTGATGACG |  | This study |
|  | RThRS2R | ACACGGGCATAGGTCAGTTC |  | This study |
|  | RThPT2R | TATCGGCGGGGACATAGATA |  | This study |
| Construction of p_hem1_::*lacZ* transcriptional fusions | hemP1F | tagaattcATTCGACCCGGTGGTTTACT | EcoRI | This study |
|  | hemP1R | taggtaccCATTCATTGTGGGTGCTTTG | KpnI | This study |
| Construction of p_hem2_::*lacZ* transcriptional fusions | hemP2F | gcgaattcCACTCCATTCACACCACTCATC | EcoRI | This study |
|  | hemP2R | atgggtaccTAAGAGGAAACGGTTTGGTCG | KpnI | This study |
| Confirmation the correctness of fusions constructed in pCM132Gm | pCM132GmSpr1 | CTGCAAGGCGATTAAGTTGG |  | Nieckarz et al., 2017 |
|  | pCM132GmSpr2 | CATAAACTGCCAGGCATCAA |  | Nieckarz et al., 2017 |
| Construction of HemR-GFP translational fusion | hemR-fw | tttggtctctGTGGGCATGGTGTCGCTTTTATCA | BsaI | This study |
|  | hemR-rev | tttggtctctTAGCCAAACTGAGTGGGGACCAA | BsaI | This study |
|  | OR181-plac-fw | tttggtctctATTCTGAGCGCAACGCAATTAATG | BsaI | Kakoschke et al., 2016 |
|  | OR181-plac-rev | tttggtctctCCACCCACACAACATACGAGCCGG | BsaI | Kakoschke et al., 2016 |
| Construction of *fur* deletion mutants | Fur1 | gctctagaGCGAAGAACTGAATCTGGCCG | XbaI | This study |
|  | Fur2 | CATCCGTTTCCACGAGTCATGCGGATTC |  | This study |
|  | Fur3 | GAATCCGCATGACTCGTGGAAACGGATG |  | This study |
|  | Fur4 | TTTATCGTGCGCAGCGATCTCGGCTTGA |  | This study |
|  | Fur5 | TCAAGCCGAGATCGCTGCGCACGATAAA |  | This study |
|  | Fur6 | cgtctagaGGCAGCGCTTTGCCAACATC | XbaI | This study |
|  | Fur0 | GCGTTGAAGCTTGGGTAAAA |  | This study |
|  | Fur7 | TTTCCGGTCAATCATGGAAT |  | This study |
| EMSA, fragment F1 | hem1-aF | CGGATTATCAAGGCAACCGA |  | This study |
|  | hem1-aR | TTTATTGTTAATTTATTTTTACTTCGCG |  | This study |
| EMSA, fragment F2 (OBS-1) | hem1F | ATTCGACCCGGTGGTTTACT |  | This study |
|  | hem1R | CATTCATTGTGGGTGCTTTG |  | This study |
| EMSA, produces fragment F3 | hem1-bF | AATCATGATTGATAATGCTTATCATATTG |  | This study |
|  | hem1R | CATTCATTGTGGGTGCTTTG |  | This study |
| EMSA, fragment OBS-2 (F4) | hem2F | CCGGTTCAATTAGCATTAACTC |  | This study |
|  | hem2R | GGTTGCATTTTGCGTTATGG |  | This study |
| EMSA, fragment of 16S rDNA used as a negative control | 16SF | TACGCATTTCACCGCTAC |  | This study |
|  | 16SR | CAGAAGAAGCACCGGCT |  | This study |
| Construction of pHEM1 | hemPR-F | gcgaattcCCTGGTTACTCGGGAAGATG | EcoRI | This study |
|  | hemPR-R | gcgaattcTTACCACTGATAGCTCACGA | EcoRI | This study |
| Construction of pHEM2 | hemPR2-F | gcgaattcGCTAAGGCTCTACTCCATCA | EcoRI | This study |
|  | hemPR2-R | gcgaattcTGATAACTGATCAGCACCTT | EcoRI | This study |

*- 5’ extensions added to the primers are shown as lowercase

**REFERENCES**

Kakoschke, T.K., Kakoschke, S.C., Zeuzem, C., Bouabe, H., Adler, K., Heesemann, J. et al. (2016). The RNA chaperone Hfq is essential for virulence and modulates the expression of four adhesins in *Yersinia enterocolitica*. Sci Rep. 6, 29275. DOI:10.1038/srep29275.

Nieckarz, M., Raczkowska, A., Jaworska, K., Stefańska, E., Skorek, K., Stosio D., Brzostek, K. (2017) The Role of OmpR in the Expression of Genes of the KdgR Regulon Involved in the Uptake and Depolymerization of Oligogalacturonides in *Yersinia* *enterocolitica*. *Front. Cell. Inf. Microbiol.* 7, 366. doi: 10.3389/fcimb.2017.00366
